# Deciphering the Functional Long Non‐Coding RNAs Derived from MicroRNA Loci

**DOI:** 10.1002/advs.202203987

**Published:** 2023-10-17

**Authors:** Weiqian Li, Yue Huo, Yue Ren, Chenxi Han, Shuo Li, Kangning Wang, Manman He, Yiying Chen, Yanran Wang, Lingjie Xu, Yuehong Guo, Yanmin Si, Yufeng Gao, Jiayue Xu, Xiaoshuang Wang, Yanni Ma, Jia Yu, Fang Wang

**Affiliations:** ^1^ State Key Laboratory of Common Mechanism Research for Major Diseases Institute of Basic Medical Sciences Haihe Laboratory of Cell Ecosystem The Key Laboratory of RNA and Hematopoietic Regulation Chinese Academy of Medical Sciences / Peking Union Medical College Beijing 100005 P.R. China; ^2^ Emergency Department of West China Hospital Sichuan University Chengdu 610041 P.R. China; ^3^ The Institute of Blood Transfusion Chinese Academy of Medical Sciences / Peking Union Medical College Chengdu 610052 P.R. China

**Keywords:** CRISPR screen, miR‐301b, miRNA gene‐originated lncRNAs (molncRNAs), SMARCA5

## Abstract

Albeit the majority of eukaryotic genomes can be pervasively transcribed to a diverse population of lncRNAs and various subtypes of lncRNA are discovered. However, the genome‐wide study of miRNA‐derived lncRNAs is still lacking. Here, it is reported that over 800 miRNA gene‐originated lncRNAs (molncRNAs) are generated from miRNA loci. One of them, molnc‐301b from *miR‐301b* and *miR‐130b*, functions as an “RNA decoy” to facilitate dissociation of the chromatin remodeling protein SMARCA5 from chromatin and thereby sequester transcription and mRNA translation. Specifically, molnc‐301b attenuates erythropoiesis by mitigating the transcription of erythropoietic and translation‐associated genes, such as *GATA1* and *FOS*. In addition, a useful and powerful CRISPR screen platform to characterize the biological functions of molncRNAs at large‐scale and single‐cell levels is established and 29 functional molncRNAs in hematopoietic cells are identified. Collectively, the focus is on miRNA‐derived lncRNAs, deciphering their landscape during normal hematopoiesis, and comprehensively evaluating their potential roles.

## Graphical abstract

This work comprehensively characterizes over 860 miRNA gene‐originated lncRNAs (molncRNAs) and clarifies their landscape during erythropoiesis. One of them, molnc‐301b, regulates erythroid differentiation independent of its cognate miRNAs by coordinating the transcription and translation efficiency of key erythroid regulators. Finally, a screening platform using CRISPR‐based single‐cell RNA sequencing is established to characterize functional molncRNAs at large‐scale and single‐cell levels.

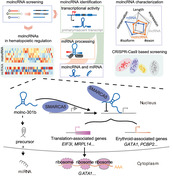

## 1 Introduction

In mammals, the transcriptional landscape is far more complicated than what we have known and what we originally imagined. Only a small proportion of the mammalian genome can be transcribed into protein‐coding mRNA, while the vast majority of them pervasively produce diverse and numerous long non‐coding RNAs (lncRNAs). Typically, lncRNAs are defined as transcripts that are longer than 200 nucleotides (nt) and without protein‐coding potential. LncRNAs exhibit a surprisingly wide range of sizes, shapes, and functions and are derived from introns or antisense transcripts of protein‐coding genes, enhancers, and other open genomic regions. Over the past decade, a wide variety of lncRNAs have been identified due to advances in transcriptome sequencing technology. For example, sno‐lncRNAs are intron‐derived lncRNAs with snoRNA caps at both ends.^[^ ^1^ ^]^ When introns with snoRNA ends are processed by snoRNA machinery and the internal sequences are not degraded, sno‐lncRNAs lacking 5′‐caps or poly(A)‐tails accumulate.^[^ ^1^ ^]^ In contrast, introns without snoRNA ends can be trimmed into circular intronic lncRNAs,^[^ ^2^ ^]^ which are generated by sequences that escape during the debranching of intron lariats.^[^ ^2^ ^]^ Natural antisense transcripts are transcribed from the opposite strands of protein‐coding or sense strands and are presumably mRNA‐like lncRNAs, which can interfere with transcription or mRNA stability.^[^ ^3^ ^]^ Additionally, the 5′‐snoRNA‐ended and 3′‐polyadenylated lncRNAs derived from read‐through transcripts sequester multiple RNA‐binding proteins in human embryonic stem cells (hESCs) and regulate alternative splicing.^[^ ^4^ ^]^ Moreover, transcripts derived from enhancers are defined as enhancer RNAs.^[^ ^5^, ^6^ ^]^

Virtually all RNA species in eukaryotes are subject to complicated post‐transcriptional processing. In particular, microRNA (miRNA) biogenesis occurs via multiple steps: nuclear processing of primary miRNA (pri‐miRNA) by the Drosha/DGCR8 complex, nuclear export of precursor miRNA (pre‐miRNA) by Exportin 5, cytoplasmic processing of pre‐miRNA by Dicer, and strand selection in the Argonaute complex.^[^ ^7^ ^]^ The studies reported by us and other colleagues have shown that miRNA processing is tightly regulated,^[^ ^8^, ^9^, ^10^ ^]^ leading to the accumulation of certain intermediates, such as pri‐miRNAs or pre‐miRNAs. Very similar to lncRNAs, the pri‐miRNAs transcribed from miRNA loci are usually >200 nt. Whereas, only the stem‐looped precursors are required to generate mature miRNAs.^[^ ^7^ ^]^ Therefore, it might be possible that these long transcripts provide the raw material for the evolution of diverse lncRNAs. Indeed, several studies have revealed the existence of such functional pri‐miRNAs, including H19 (processed from the host gene of miR‐675), which play important roles in different biological processes.^[^ ^11^, ^12^ ^]^ In addition, linc‐MD1 (processed from the host gene of miR‐133b) has been shown to regulate muscle differentiation by acting as a ceRNA in mouse and human myoblasts^[^ ^13^ ^]^ and LEADeR (processed from the host gene of miR‐205) orchestrates human prostate basal luminal differentiation.^[^ ^14^ ^]^ Moreover, Chang et al. developed an approach to annotate genomic locations of pri‐miRNAs from RNA sequencing (RNA‐seq) data,^[^ ^15^ ^]^ while Dhir et al. depicted a novel transcription termination mechanism of lncRNA genes that host miRNAs (lnc‐pri‐miRNAs)^[^ ^16^ ^]^ and Daniel found that one of the functional lnc‐pri‐miRNA loci, *LOC646329/MIR29HG*, could regulate glioblastoma multiforme cell growth independent of its cognate miRNA.^[^ ^17^ ^]^ However, it's still unknown whether there are any lncRNAs derived from the vast majority of other miRNA loci, and how these miRNA gene‐originated lncRNAs (molncRNAs) exert their biological function in normal bioprocess.

Here, we identified >860 molncRNAs from 303 annotated human miRNA loci and clarified a global molncRNA landscape during hematopoiesis. Among the molncRNAs identified, molnc‐301b coordinated the expression of key erythroid regulators at both transcriptional and translational levels to attenuate erythropoiesis either in human hematopoietic stem cells (HSCs) or ESCs, and such effect is independent of its two cognate miRNAs, miR‐130b and miR‐301b. Finally, we combined pooled CRISPR‐perturbation with single‐cell transcriptome sequencing to screen the potential functional molncRNAs on a genome‐wide scale and identified 29 functional molncRNAs.

## 2 Results

### 2.1 Identification of molncRNAs by Single‐Molecule Long‐Read Sequencing in Human Hematopoietic Cells

**Figure 1:**
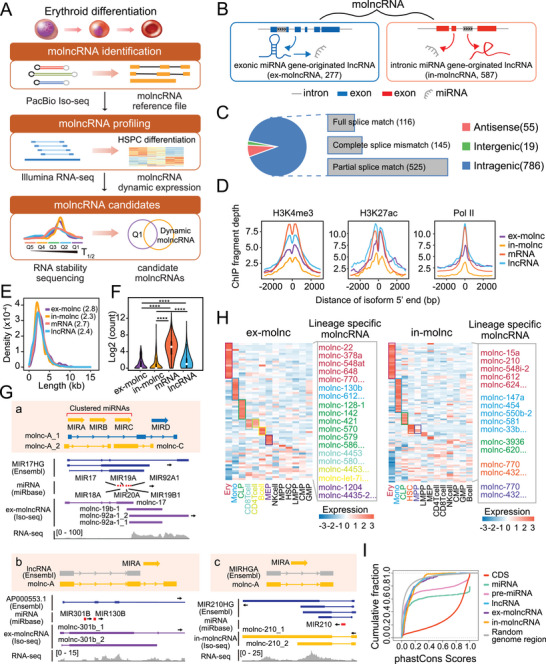
Identification and characterization of molncRNAs. A) Schematic illustration of molncRNA identification and characterization. B) MolncRNAs were divided into two subtypes, ex‐molncRNAs (277) and in‐molncRNAs (587), based on whether mature miRNAs are embedded in the exonic or intronic sequences of their host molncRNAs. C) Proportion of molncRNAs located in different gene regions. D) Metaplot showing the distribution of H3K4me3, H3K27ac, and Pol II ChIP‐seq fragment depth within −3000 to 3000 bp centered around the 5′ end of molncRNA, mRNA, and lncRNA based on PacBio Iso‐seq (*n* = 2 replicates). E) Length distribution of molncRNA, mRNA, and lncRNA based on PacBio Iso‐seq. The median length is shown in brackets. F) Expression levels of molncRNA, mRNA, and lncRNA, determined by PacBio Iso‐seq. *p*‐Values were calculated by a two‐sided Mann‐Whitney test. ****p <0.0001. G) Schematic illustration and integrative Genomics Viewer (IGV) plot of three kinds of relations among annotated lncRNAs, pre‐miRNAs, and molncRNAs. The reference sequence in Ensembl and miRbase, full‐length molncRNAs generated by Iso‐seq and corresponding mapped short reads in poly(A)^+^ RNA‐seq are displayed in order from top to bottom in IGV plot. The thick lines represent exons and the thin lines represent introns. The arrows represent the transcription orientation. H) Heatmap of specific molncRNA expression across 13 distinct hematopoietic lineages (*n* = 2 replicates). I) Evolutionary conservation analysis of molncRNA and other annotated genomic elements based on phastCons scores.

To determine the dynamic expression of molncRNAs during erythropoiesis, we employed a multi‐step strategy combining PacBio Iso‐seq with Illumina short‐read RNAseq and RNA stability assays of differentiating hematopoietic cells (**Figure** **1**A).

The PacBio Iso‐seq of K562 cells, which represent a robust model for studying erythroid gene expression in vitro, yielded 41909348 high‐quality subreads, from which 1221669 full‐length non‐chimeric (FLNC) reads were identified after poly(A) tail and concatemer removal. We then identified a high‐quality set of 434485 non‐redundant PacBio transcripts (see Experimental Section), including 12005 annotated mRNAs (286092 transcripts) and 2194 lncRNAs (10789 transcripts) (Figure S1A; Table S1, Supporting Information).

Subsequently, we generated the molncRNA set (860 transcripts), defined as any long PacBio isoform (>200 nt) overlapping with a pre‐miRNA on the same strand, with no detectable protein‐coding potential and no splicing sites shared with annotated protein‐coding genes (see Experimental Section). Moreover, based on the location of miRNAs in the exonic or intronic region of their host genes, molncRNAs were divided into 2 subtypes, exonic miRNA gene‐originated lncRNA (ex‐molncRNA; *n* = 277) and intronic miRNA gene‐originated lncRNA (in‐molncRNA; *n* = 587) (Figure 1B). Notably, because some molncRNAs (*n* = 4) cover different miRNAs, they might be classified as ex‐ or in‐molncRNA. We subsequently compared the identified molncRNAs with annotated genes (Figure 1C, Figure S1B, Supporting Information) and classified them into three categories: i) 55 molncRNAs that derived from the antisense strands of an annotated gene (e.g., molnc‐3124; Figure S1C, Supporting Information, upper panel); ii) 19 molncRNAs that derived from the intergenic region (e.g., molnc‐3685; Figure S1C, Supporting Information, lower panel), and iii) 786 molncRNAs that are located in the intragenic region, which are further classified into three subcategories based on their shared spliced sites with annotated gene (Figure 1C; Figure S1B, Supporting Information): full splice match (FSM), complete splice mismatch (CSM) and partial splice match (PSM). Among them, 116 FSM molncRNAs are identical to the annotated lncRNAs; 145 CSM molncRNAs have completely different splicing junctions to known genes, which are unannotated lncRNAs derived from the same genic loci; the remaining 525 PSM molncRNAs partially share splice sites with known genes, which might be different isoforms of known lncRNAs (Figure 1C; Figure S1B, Supporting Information).

Similar to actively transcribed mRNAs and lncRNAs, the promoter regions of molncRNAs are also enriched with active markers such as H3K4 tri‐methylation (H3K4me3), H3K27 acetylation (H3K27ac) modifications and Pol II signals (Figure 1D). The majority of molncRNAs ranged in length from 1800 to 3500 nt (25%–75%), which was similar to both the mRNAs and classic lncRNAs captured by PacBio Iso‐seq (Figure 1E). Nevertheless, like classic lncRNAs, the molncRNAs were expressed at much lower levels than mRNAs (Figure 1F), especially the in‐molncRNAs. Unlike protein‐coding genes and classic lncRNAs, molncRNAs were generally generated as a unique transcript (Figure S1D, Supporting Information), suggesting that they are derived from relatively fixed transcription units and are less commonly processed by alternative splicing. Besides, most of the ex‐molncRNAs had only one exon, whereas in‐molncRNAs had a comparable number of exons to mRNAs and even more than classic lncRNAs (Figure S1E, Supporting Information). Thus, ex‐molncRNAs exist mainly as intronless genes, which have been reported to show stronger tissue specificity and are often associated with signal transduction (e.g., G protein‐coupled receptors), transcriptional regulation (e.g., histones), and other biological functions.^[^ ^18^, ^19^ ^]^

### 2.2 Characterization of molncRNA

To systematically characterize molncRNAs, we also performed small RNA‐seq. We found that half of the annotated miRNAs (*n* = 980) were detected in hematopoietic cells, of which 567 were located in an annotated coding gene region and 169 were located in ncRNA region (Figure S1F, Supporting Information). The aforementioned 277 ex‐molncRNAs and 587 in‐molncRNAs transcripts were derived from 303 annotated human miRNA genes (138 exonic and 204 intronic), accounting for approximately 16% (303 of 1881) of the total miRNA genes (miRBase v21). Among these, 78% (215 of 277) ex‐molncRNA (105 miRNAs) and 78% (455 of 587) in‐molncRNA (147 miRNAs) transcripts were co‐detected with their corresponding mature miRNAs. Of note, corresponding miRNAs were not detected for 62 ex‐molncRNAs and 132 in‐molncRNAs, further indicating the possibility that molncRNAs fulfill independent roles. In addition, we can't detect any molncRNAs from other 1578 miRNAs, which might be due to the rapid processing of their primary transcripts to generate mature miRNAs (Figure S1F; Table S1, Supporting Information). Moreover, miRNAs co‐expressed with molncRNAs showed higher expression levels than those derived from non‐molncRNA‐detected loci (Figure S1G; Table S1, Supporting Information), which might be due to a higher transcription activity of both *ex‐* or *in‐molncRNA* loci favoring the output of both molncRNAs and miRNAs (Figure S1H, Supporting Information). In addition, we found that miRNAs tended to be enriched at the 3′ end of in‐molncRNA loci and were evenly distributed in ex‐molncRNA loci (Figure S1I, Supporting Information).

Interestingly, the comparison between molncRNA sequences and their cognate miRNAs revealed some new information regarding either miRNA clusters or their neighbor lncRNA genes (Figure 1G; Table S1, Supporting Information). a) The revision of the defined miRNA cluster, which means that the actual miRNA cluster is either beyond or less than the current definition in miRbase v21. Distant miRNAs may be located on the same transcription unit. For example, miR‐6724‐2, a miRNA 40 kb far from the miR‐3648‐1–3687‐1 cluster locus, was also restricted to molnc‐3648‐1. In addition, clustered miRNAs might be derived from different pri‐miRNAs, such as miR‐17–92 cluster. Four miR‐17–92 cluster‐derived molncRNAs were identified. Among them, molnc‐17 was presumed to contain all six miRNAs and molnc‐19b‐1 covered only miR‐19b‐1 and −92a‐1, while the two molnc‐92a‐1 isoforms were specific to miR‐92a‐1. It is worth noting that molnc‐19b‐1 and molnc‐92a‐1 were also supported by RNA‐seq short reads (Figure 1G). b) The redefinition of existing lncRNA, such as molnc‐301b, a novel transcript which was distinct from the annotated lncRNA AP000553.1 (Figure 1C,G). c), The validation of the reported miRNA host gene. For example, the molnc‐210 splicing site matched with MIR210HG transcript (Figure 1G).

To further develop an overview of the tissue‐ or lineage‐specificity of molncRNAs, we compared their expression profiles with published RNA‐seq datasets. Here, to avoid confusion with known protein‐coding genes, molncRNAs overlapping by >500 nt were excluded, thereby yielding a dataset consisting of 239 ex‐molncRNAs and 557 in‐molncRNAs (Figure S1J, Supporting Information). PacBio Iso‐seq was first used to construct the molncRNA reference sequences for RNA‐seq read mapping (Figure S1K, Supporting Information). We then constructed a comprehensive atlas of molncRNA expression across 16 human tissues by comparison with the Human BodyMap 2.0 project.^[^ ^20^ ^]^ Stingingly, we found that 45% of ex‐molncRNAs and 30% of in‐molncRNAs displayed tissue‐specific expression. These included the white blood cell‐specific ex‐molnc‐223, −128‐1, and −558, and the testes‐specific in‐molnc‐379, −432, and −15a (Figure S1L; Table S2, Supporting Information). Subsequently, the hematopoietic expression atlas was established in 13 distinct blood cell populations.^[^ ^21^ ^]^ We found that 64% of ex‐molncRNAs and 67% in‐molncRNAs showed lineage‐specific accumulation, such as the top‐ranked erythrocyte‐specific molncRNAs ex‐molnc‐22, −378a and in‐molnc‐15a, and −210 (Figure 1H; Table S2, Supporting Information).

We also assessed the conservation of molncRNA based on phastCons scores across 100 species by comparison with different genomic elements. The molncRNAs were less conserved than dominant mature miRNAs, pre‐miRNAs, and CDSs, while similar to the lower conservation of classic lncRNAs (Figure 1I). Thus, we speculated that the primary route of functional lncRNA evolution is from junk transcripts corresponding to the “transcription first” model.^[^ ^22^ ^]^

### 2.3 Dynamic molncRNA Expression During Human Hematopoiesis

**Figure 2:**
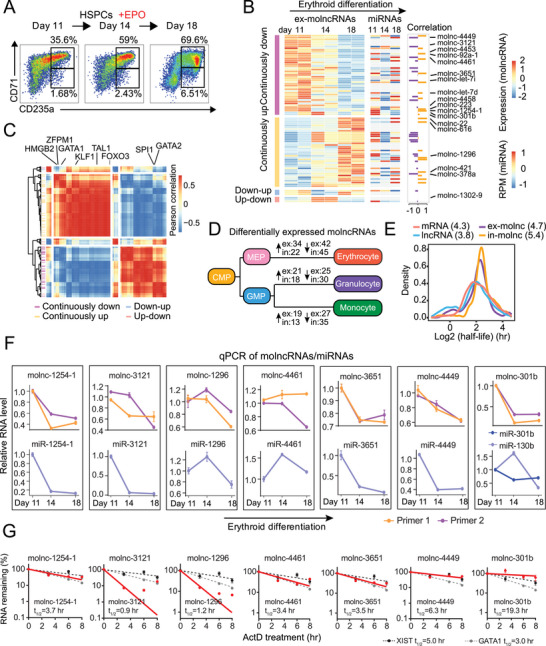
MolncRNAs have long half‐lives and show dynamic changes during human hematopoiesis. A) Flow cytometric analysis of erythroid surface markers CD71 and CD235a. The percentage of CD71^high^/CD235a^+^ and CD71^low^/CD235a^+^ cells are indicated. B) Heatmap of differentially expressed ex‐molncRNAs (left panel, *n* = 2 replicates) and their cognate miRNAs (middle panel) during erythroid differentiation. Pearson correlation analysis of molncRNAs and their cognate miRNAs is displayed in the right panel. C) Pearson correlation analysis (*n* = 2 replicates) of ex‐molncRNAs and critical erythroid regulators (Table S3, Supporting Information) collected from the GO database using the following keywords: hematopoiesis, hematopoietic, and erythrocyte. The expression tendency of molncRNAs is shown on the left. D) Number of ex‐ and in‐molncRNAs differentially expressed during human hematopoiesis. Arrows indicate continuously up‐ or down‐regulated molncRNAs. E) Half‐life distribution of molncRNA, mRNA, and lncRNA in erythrocytes. The median half‐lives are shown in brackets (*n* = 2 replicates). F) qPCR analysis of the relative expression levels of selected ex‐molncRNAs and their cognate miRNAs during HSPC erythroid differentiation. Two primer pairs were used for each molncRNA (primers 1 and 2). Data represent the mean ± SD (*n* = 3 replicates). G) Decay curves of seven candidate ex‐molncRNAs in K562 cells. LncRNA XIST and *GATA1* mRNA were used as controls. Data represent the mean ± SD (*n* = 3 replicates).

To further investigate the potential function of molncRNAs, we induced erythroid differentiation of CD34^+^ hematopoietic stem/progenitor cells (HSPCs) in vitro. Illumina poly(A)^+^ RNA‐seq and small RNA‐seq were performed to profile the expression of molncRNAs and their corresponding miRNAs, respectively (**Figure** **2**A; Figure S2A,B, Supporting Information). We identified 82 ex‐molncRNAs and 75 in‐molncRNAs showing dynamic expression during erythropoiesis (Figure 2B; Figure S2C, Supporting Information). In addition, analysis of our previous RNA‐seq datasets of monocytic and granulocytic differentiation^[^ ^9^ ^]^ revealed that 22 molncRNAs showed a consistent decrease (e.g., ex‐molnc‐3121, −4449, −4461 and in‐molnc‐4453‐2) or increase (e.g., ex‐molnc‐616) in expression during erythroid, monocytic and granulocytic differentiation. Furthermore, the expression of 39 molncRNAs changed only during erythroid differentiation (e.g., decreased expression of ex‐molnc‐1254‐1, in‐molnc‐1207, −3159 or increased expression of ex‐molnc‐421), and 30 molncRNAs displayed opposite changes between erythroid and the other two myeloid lineages (e.g., decreased expression of ex‐molnc‐let‐7d, ‐let‐7i, and −223 or increased expression of ex‐molnc‐548c and in‐molnc‐6739 in erythropoiesis) (Figure 2B,D; Figure S2C,D; Table S3, Supporting Information). We also compared the changes in the expression of molncRNAs with their cognate miRNAs during erythropoiesis. Pearson correlation analysis revealed that 42 pairs showed a significantly high correlation. Of these, 22 exhibited changes consistent with those of miRNAs (e.g., ex‐molnc‐223, −616, −22, and in‐molnc‐374b), and 20 showed inverse changes in expression compared with miRNAs (e.g., ex‐molnc‐let‐7i, −4449, −378a, and in‐molnc‐3157) (Figure 2B; Figure S2C,E, Supporting Information).

Since ex‐molncRNAs contain miRNA sequences, they are more likely to serve as pri‐miRNAs. Therefore, to specifically evaluate pri‐miRNAs as a new type of lncRNA, we then focused mainly on ex‐molncRNAs. To predict the involvement of ex‐molncRNAs in erythropoiesis, we analyzed the correlation of their expression with critical erythroid genes defined by hematopoiesis‐related terms from the Gene Ontology (GO) database^[^ ^23^ ^]^ (Table S3, Supporting Information). We found that upregulated ex‐molncRNAs correlated positively with essential erythroid activators (e.g., *GATA1*, *KLF1*, *TAL1*, and *FOXO3*) and negatively with erythroid repressors (e.g., *GATA2* and *SPI1*), while downregulated ex‐molncRNAs exhibited the opposite correlations (Figure 2C), indicating the potential roles of these ex‐molncRNAs.

### 2.4 Analysis of molncRNA Stability During Human Erythroid Differentiation

To further screen functional molncRNAs, we performed RNA stability profiling to evaluate their half‐life (Figure S2F, Supporting Information). Interestingly, molncRNAs had longer half‐lives than annotated mRNAs and classic lncRNAs (Figure 2E; Figure S2G; Table S3, Supporting Information), providing compelling evidence for the functional significance of molncRNAs. RNAs were divided into five intervals (Q1–Q5) based on mRNA half‐life quintile ranked from the longest to shortest (Figure S2H; Table S3, Supporting Information). Compared with annotated mRNAs and lncRNAs, molncRNAs were more commonly distributed in the relatively long half‐life intervals (Q1 and Q2).

By combining the dynamic expression profiles with RNA stability data, we selected seven ex‐molncRNAs (molnc‐1254‐1, −3121, −1296, −4461, −3651, −4449 and molnc‐301b) belonging to the most stable Q1 interval for further analysis (Figure S2I, Supporting Information). Molnc‐4449, −4461, and −3121 were downregulated during monocytic and granulocytic differentiation, while the expression of the others was unchanged or showed irregular changes (Figure S2D, Supporting Information). Consistent with RNA‐seq, we verified the down‐regulation of molnc‐1254‐1, −301b, −4449, −3651 and −3121 during HSPC erythroid differentiation, and the changes in the expression of most miRNAs were in line with those of the molncRNAs (Figure 2F). Of note, molnc‐301b and miR‐301b‐3p were downregulated during HSPC erythroid differentiation, while the changes in miR‐130b‐3p expression were irregular. Moreover, qPCR analysis of RNA half‐lives showed that molnc‐301b was more stable than other molncRNAs, even *GATA1* mRNA and lncRNA XIST (Figure 2G). Based on these results, we selected molnc‐301b for further investigation of its function during erythroid differentiation.

### 2.5 Molnc‐301b Regulates Erythroid Differentiation Independent of its Cognate miRNAs

**Figure 3:**
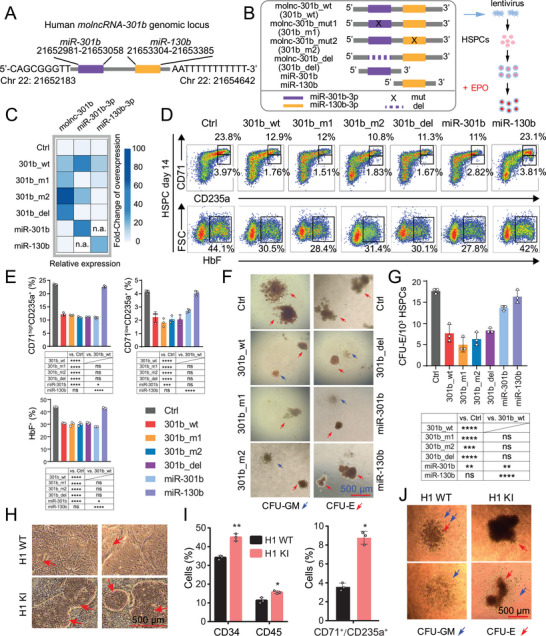
Molnc‐301b regulates erythroid differentiation independent of its cognate miRNA. A) Genomic locus of the molnc‐301b transcript determined in RACE assays. B) Schematic illustration of a series of 301b constructs (301b_wt, 301b_m1, 301b_m2, 301b_del, miR‐301b and miR‐130b. These vectors were used to generate lentiviruses to infect HSPCs. C) Heatmap showing the relative expression levels of molnc‐301b, miR‐301b, and miR‐130b in a series of molnc‐301b construct‐overexpressing HSPCs. n.a., not available. D) Representative flow cytometry plots of CD71^high^/CD235a^+^, CD71^low^/CD235a^+,^ and HbF^+^ cells in empty vector‐ (Ctrl) or molnc‐301b construct‐overexpressing HSPCs at day 14 during erythroid differentiation. E) Percentage of CD71^high^/CD235a^+^, CD71^low^/CD235a^+,^ and HbF^+^ cells in Ctrl‐ or molnc‐301b construct‐overexpressing HSPCs after day 14 of erythroid differentiation detected by flow cytometry. Data represent the mean ± SD (*n* = 3 replicates). *p*‐Values were calculated by one‐way ANOVA. *p <0.05, ***p <0.001, ****p <0.0001, ns, not significant. F) Colony‐forming unit assay of Ctrl‐ or molnc‐301b construct‐overexpressing HSPCs. Red arrows indicate colony‐forming unit‐erythroid (CFU‐E) and blue arrows indicate colony‐forming unit‐granulocyte, macrophage (CFU‐GM). Scale bar, 500 µm. G) Average frequency of colony formation per 10^3^ HSPCs. Data represent the mean ± SD (*n* = 3 replicates). *p*‐Valuess were calculated by unpaired one‐way ANOVA. **p <0.01, ***p <0.001, ****p <0.0001, ns, not significant. H) Representative morphology of “cobblestone‐like” cells generated from H1 WT and molnc‐301b H1 KI hESCs at day 9 of hematopoietic differentiation. The “cobblestone‐like” cells are indicated by red arrows. Scale bar, 500 µm. I) Left: percentage of CD34^+^ and CD45^+^ cells in H1 WT and H1 KI hESCs on day 4 of suspension culture. Right: percentage of CD71^+^/CD235a ^+^ cells on day 4 of erythroid differentiation detected by flow cytometry. Data represent the mean ± SD (*n* = 3 replicates). *p*‐Valuess were calculated by unpaired *t*‐test. *p < 0.05, **p <0.01. J) Colony‐forming unit assay of H1 WT and HI KI hESCs. Red arrows indicate CFU‐E and blue arrows indicate CFU‐GM. Scale bar, 500 µm.

To investigate the function of molnc‐301b, we first used 5′ and 3′ rapid‐amplification of cDNA ends (RACE) to obtain the accurate transcript of molnc‐301b in K562 cells (Figure S3A, Supporting Information). We obtained a 2,460 nt transcript with a poly(A) tail, which was nearly (identity: 99.53%) matched with the sequences captured by PacBio Iso‐seq. Thus, we concluded that the human *molnc‐301b* locus was located on chromosome 22 and comprised two annotated miRNA genes (miRBase v21): *miR‐301b* and *miR‐130b* (**Figure** **3**A). To further determine the involvement of molnc‐301b in erythropoiesis and distinguish it from the mature miRNAs, we constructed a series of molnc‐301b mutants comprising miR‐301b‐3p or miR‐130b‐3p seed sequence mutants (301b_m1 and 301b_m2), a miR‐301b‐3p deletion mutant (301b_del) and two vectors expressing only the mature miRNAs (miR‐301b and miR‐130b). As expected, the wild‐type molnc‐301b (301b_wt) generated both molnc‐301b and the two mature miRNAs, while 301b_m1 and 301b_m2 expressed molnc‐301b but no mutant miRNAs and miR‐301b and miR‐130b generated only the miRNAs (Figure 3B,C; Figure S3B, Supporting Information). Of note, the 301b_del expressed only molnc‐301b but none of the two mature miRNAs (Figure 3C and S3B, Supporting Information), which might be due to the disruption of pri‐miRNA structure caused by sequence deletion.

We transduced these constructs into HSPCs (Figure 3B) to evaluate their effects on erythroid differentiation. Flow cytometric analysis revealed that with the exception of miR‐130b, overexpression of either wild‐type or mutant molnc‐301bs uniformly decreased the percentage of differentiated erythroid cells (CD71^high^/CD235a^+^ and CD71^low^/CD235a^+^) and globin‐positive (HbF^+^) cells (Figure 3D,E; Figure S3C, Supporting Information). CFU assays also showed consistent repression of CFU‐Es following the introduction of all molnc‐301bs except miR‐130b (Figure 3F,G). Among these molnc‐301b constructs, 301_del, which generates only molnc‐301b, still inhibited erythropoiesis, thus indicating its independent role in this process. In contrast, loss‐of‐function analysis showed that molnc‐301b reduction (Figure S3D, Supporting Information) significantly promoted the percentage of differentiated erythroid cells (CD71^high^/CD235a^+^ and CD71^low^/CD235a^+^) and HbF^+^ cells (Figure S3E,F, Supporting Information), significantly increased the number of CFU‐Es (Figure S3G,H, Supporting Information). Collectively, our findings show that molnc‐301b regulates erythroid differentiation independent of its cognate mature miRNAs, suggesting that different transcripts (molnc‐301b, miR‐310b, and miR‐130b) derived from the same genomic locus play divergent functions.

Besides CD34^+^ HSPCs, we employed a hESC differentiation system to genetically confirm the impact of molnc‐301b on erythropoiesis. Using a CRISPR/Cas9 genome editing strategy, we generated the miR‐301b‐4×poly(A) knockin H1 ESC (H1 KI), in which a 4×poly(A) sequence was knocked into the sequence downstream of the molnc‐301b transcription start site (TSS) (Figure S3I, Supporting Information). To eliminate confusion with mature miR‐301b, we also incorporated a miR‐301b precursor sequence upstream of the 4×poly(A) to maintain miR‐301b expression after molnc‐301b termination (Figure S3I,J, Supporting Information). As expected, molnc‐301b transcription was suppressed in H1 KI cells compared with the wild‐type H1 (H1 WT) cells. Nevertheless, mature miR‐301b expression was not disrupted (Figure S3K, Supporting Information).

Subsequently, we subjected the H1 WT and H1 KI ESCs to a multi‐stage erythroid induction process.^[^ ^24^ ^]^ Molnc‐301b deficiency promoted the generation of cobblestone‐like cells during hematopoietic differentiation (Figure 3H). Compared to H1 WT cells, an increase in the proportion of CD34^+^/CD45^+^ hematopoietic cells was observed in H1 KI ESCs. Moreover, CD71^+^/CD235^+^ erythroid cells were increased when the expression of molnc‐301b was terminated (Figure 3I). In accordance with the flow cytometry results, we detected more CFU‐Es derived from H1 KI ESCs compared with the number derived from H1 WT cells (Figure 3J; Figure S3L, Supporting Information). Collectively, this hESC‐based genetic system further characterizes and validates the essential biological function of molnc‐301b during erythropoiesis.

### 2.6 Molnc‐301b Acts as a “Decoy” to Promote the Dissociation of the Chromatin Remodeling Protein SMARCA5 from Chromatin

**Figure 4:**
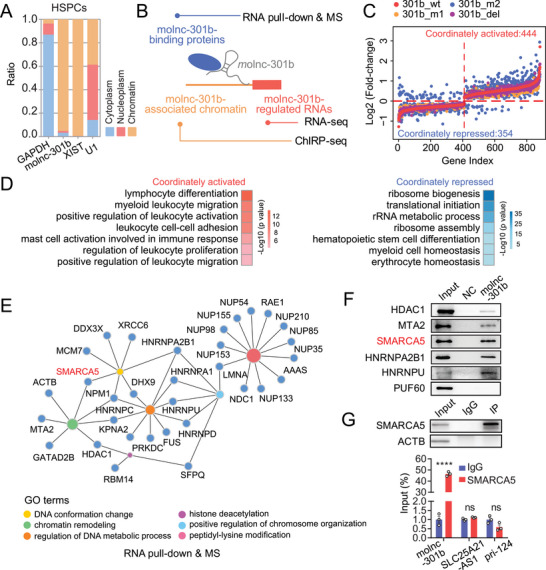
Molnc‐301b interacts with SMARCA5. A) Fractions of RNAs located in the chromatin, nucleoplasm, and cytoplasm of HSPCs. *GAPDH*, *U1*, and XIST RNAs were used as positive controls for cytoplasm, nucleoplasm, and chromatin location, respectively (*n* = 3 replicates). B) Experimental design of the molnc‐301b mechanism study. C) Scatterplots showing DEGs of 301b_wt‐, 301b_m1‐, 301b_m2‐, 301b_del‐overexpressing HSPCs compared with the control. Significantly up‐ or downregulated genes were determined by *p* <0.05 (*n* = 2 replicates). D) GO functional enrichment analysis of coordinately activated and coordinately repressed genes. E) GO functional network showing chromatin‐related terms of molnc‐301b pull‐down nuclear proteins in MS analysis. F) Western blot validation of proteins associated with molnc‐301b in K562 cells. NC refers to proteins pulled down by magnetic beads. G) SMARCA5 RIP assay of K562 cells. Western blot showing SMARCA5 immunoprecipitation (upper panel). The relative fold enrichment of molnc‐301b using SMARCA5 compared with IgG was determined by qPCR analysis (lower panel). SLC25A21‐AS1 and pri‐124 transcripts were used as negative controls. Data represent the mean ± SD (*n* = 3 replicates). *p*‐Valuess were calculated by unpaired *t‐*test. ****p <0.0001, ns, not significant.

In order to determine the molecular mechanism of molnc‐301b, we first analyzed its subcellular localization and observed that molnc‐301b is predominantly associated with chromatin in HSPCs and K562 cells (**Figure** **4**A; Figure S4A, Supporting Information), implying its regulation at the chromatin interface. Subsequently, we employed a multi‐omics strategy to identify its target genes. We used RNA‐seq following molnc‐301b overexpression to identify the RNAs regulated by molnc‐301b, defined its binding proteins by RNA pull‐down combined with mass spectrometry (MS) experiment, and determined its associated genomic loci via chromatin isolation by RNA purification and sequencing (ChIRP‐seq) (Figure 4B).

Based on RNA‐seq data from 301b_wt‐, 301b_m1‐, 301b_m2‐ and 301b_del‐overexpressing HSPCs, we identified 798 differentially expressed genes (DEGs; 444 upregulated and 354 downregulated) (Figure 4C; Table S4, Supporting Information). Notably, the global perturbation of the transcriptome following transduction with wild‐type and mutant molnc‐301bs was largely (90%) consistent, supporting its independent functions. Moreover, the 444 genes coordinately activated by molnc‐301b, which represent a gene set restrictively regulated by molnc‐301b, were enriched mainly in lymphocytes and immune pathways, such as lymphocyte differentiation, myeloid leukocyte migration, and lymphocyte activation. Interestingly, the genes coordinately repressed by molnc‐301b were over‐represented in translation‐associated pathways (e.g., ribosome biogenesis, translation initiation, and ribosome assembly). Furthermore, in accordance with the function of molnc‐301b, genes associated with myeloid cell homeostasis, erythrocyte homeostasis, and HSC differentiation pathways were also downregulated (Figure 4D; Table S4, Supporting Information).

Given the fact that the vast majority of molnc‐301b is located in the nuclei, but not the cytosol, we mainly focused on the 96 nucleus‐specific proteins identified by our RNA pull‐down assays coupled MS for further study (Figure S4B; Table S4, Supporting Information). Further, molnc‐301b binds mainly to chromatin, we focused on six biological processes related to chromatin function: DNA conformation change, chromatin remodeling, regulation of DNA metabolic process, histone deacetylation, positive regulation of chromosome organization and peptidyl‐lysine modification (Figure 4E; Table S4, Supporting Information). Among the molnc‐301b‐associated proteins, SMARCA5 is a member of the SWI/SNF chromatin remodeling complex and plays indispensable roles during early hematopoiesis and erythropoiesis. Loss of SMARCA5 abrogates definitive hematopoiesis within the fetal liver and leads to anemia and embryonic lethality at day 18.5.^[^ ^25^ ^]^ The interaction between molnc‐301b and SMARCA5 were validated through RNA pull‐down assays (Figure 4F). Molnc‐301b was also specifically enriched from SMARCA5 immunoprecipitates by reciprocal immuno‐precipitation (Figure 4G).

**Figure 5:**
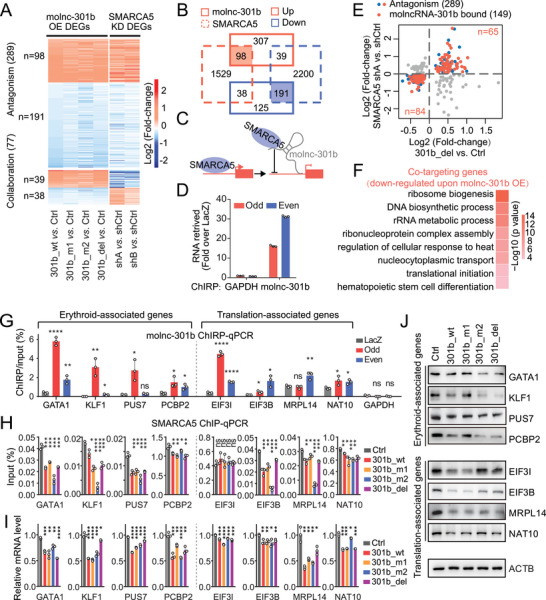
Molnc‐301b acts as an “RNA decoy” by promoting the dissociation of SMARCA5 from chromatin. A) Heatmap showing DEGs of molnc‐301b overexpression (301b_wt, 301b_m1, 301b_m2 and 301b_del) and SMARCA5 knockdown. Significantly up‐ or downregulated genes were determined by p<0.05 (*n* = 2 replicates). B) Overlapping DEGs of molnc‐301b overexpression (301b_wt, 301b_m1, 301b_m2 and 301b_del) and SMARCA5 knockdown. The colored boxes and line types denote gene expression events upregulated (red box) or downregulated (blue box) by molnc‐301b (solid line) or SMARCA5 (dashed line). C) Schematic illustration of molnc‐301b promoting the dissociation of SMARCA5 from chromatin and regulating transcription. D) qPCR validation of molnc‐301b in the immunoprecipitation products of ChIRP assay. GAPDH was used as a negative control. E) Scatterplot showing the intersection (red dots, *n* = 149) between molnc‐301b & SMARCA5 antagonistic (red and blue dots, *n* = 289) and molnc‐301b‐bound targets revealed by ChIRP‐seq. The overlapping DEGs with log2(fold‐change) of 301_del versus Ctrl and knockdown SMARCA5 shA versus shCtrl are illustrated. F) GO functional enrichment analysis of the molnc‐301b and SMARCA5 co‐targeting genes. G) ChIRP‐qPCR validation of chromatin interaction of molnc‐301b with four erythroid‐associated and another four translation‐associated gene loci. GAPDH locus was used as a negative control. H) SMARCA5 ChIP‐qPCR validation of four erythroid‐associated and four translation‐associated gene loci. I) qPCR validation of four erythroid‐associated and four translation‐associated genes in HSPCs. J) Western blot validation of four erythroid‐associated and four translation‐associated genes. For Figure 5G‐I, data represent the mean ± SD (n = 3 replicates). p‐Valuess were calculated by unpaired t‐test. *p < 0.05, **p <0.01, ***p <0.001, ****p <0.0001, ns, not significant.

Next, to elucidate the downstream effects after the association of molnc‐301b with SMARCA5, we compared the DEGs induced by SMARCA5 knockdown with those regulated by molnc‐301b overexpression. Two SMARCA5‐specific shRNAs (shA and shB) were used to suppress its endogenous expression (Figure S5A,B; Table S4, Supporting Information). Comparative analysis indicated that 366 genes were cooperatively regulated by molncRNA‐301b and SMARCA5 (**Figure** **5**A). Among them, nearly 80% (289 genes) showed consistent changes (Figure 5A,B; Table S4, Supporting Information), suggesting an antagonistic relationship between molnc‐301b and SMARCA5. These findings indicate that molnc‐301b acts as a “decoy” by binding to SMARCA5 and disrupting its association with chromatin in hematopoietic cells (Figure 5C).

To further reveal the DNA targets of molnc‐301b, we performed chromatin isolation by RNA purification (ChIRP) of molnc‐301b with antisense oligos tiled along the entire molnc‐301b transcript sequence. Both “even” and “odd” probe sets yielded comparable enrichment of expected molnc‐301b sites over *GAPDH* (Figure 5D). Deep sequencing analysis of the retrieved chromatin fragments revealed that molnc‐301b binding sites were localized mainly in genic regions. Furthermore, molnc‐301b was significantly enriched in the promoter, exon, and TTS regions relative to the whole genome, suggesting its potential roles in regulating gene transcription (Figure S5C, Supporting Information).

To determine the target genes co‐regulated by molnc‐301b and SMARCA5, we compared their antagonistic targets with molnc‐301b‐occupied genes revealed by ChIRP‐seq and found that 149 of 289 genes were bound by molnc‐301b (Figure 5E; Table S4, Supporting Information). As SMARCA5 has been reported to promote chromatin remodeling and activate gene transcription,^[^ ^26^ ^]^ we focused mainly on genes that were positively regulated by SMARCA5; that is, the genes that were negatively controlled by molnc‐301b due to their antagonistic relationship (defined as “co‐targeting genes”). GO enrichment analysis showed that the molnc‐301b and SMARCA5 co‐targeting genes were enriched in ribosome biogenesis, ribonucleoprotein complex assembly, and HSC differentiation (Figure 5F), of which the enrichment of RNA translation‐related pathways attracted our attention because previous studies have shown that reduced translation of certain transcripts in HSPCs specifically impairs erythroid lineage commitment.^[^ ^27^, ^28^ ^]^ Supporting this view is the observation that the majority of Diamond–Blackfan anemia cases are caused by heterozygous loss‐of‐function mutations of ribosomal proteins, with selective perturbation of their erythroid lineages.^[^ ^27^ ^]^ Therefore, among the co‐targeting genes, we selected the key erythroid regulators, including *GATA1*, *KLF1*, *PUS7*, and *PCBP2*, and translation‐associated genes, including *EIF3I*, *EIF3B*, *MRPL14*, and *NAT10*, for validation.

Peak calling showed enrichment of the high‐confidence molnc‐301b ChIRP‐seq peaks on these target genes (Figure S5D, Supporting Information). ChIRP‐qPCR confirmed that molnc‐301b is bound strongly to most of the target genes, such as *GATA1* and *NAT10* (Figure 5G). The ChIP‐qPCR analysis showed that SMARCA5 enrichment on these genes (except *EIF3I*) decreased following overexpression of either wild‐type or mutant molnc‐301bs (Figure 5H). As expected, the transcription of all selected target genes was repressed (Figure 5I), verifying the transcriptional repression effects of molnc‐301b on these genes. Furthermore, the protein levels of most of the target genes were also reduced (Figure 5J; Figure S5E, Supporting Information). Besides, we have performed an EMSA to detect whether the association of molnc‐301b plays a role in segregating SMARCA5 from chromatin. Results showed that molnc‐301b RNA could reduce the affinity of SMARCA5 on target DNA (Figure S5F, Supporting Information). Overall, these data suggest that molnc‐301b, acting as a “decoy”, antagonizes the function of SMARCA5 by attenuating its chromatin binding activity and thereby repressing gene transcription in hematopoietic cells.

### 2.7 Molnc‐301b Orchestrates Protein Synthesis by Controlling Translation‐Associated Genes

**Figure 6:**
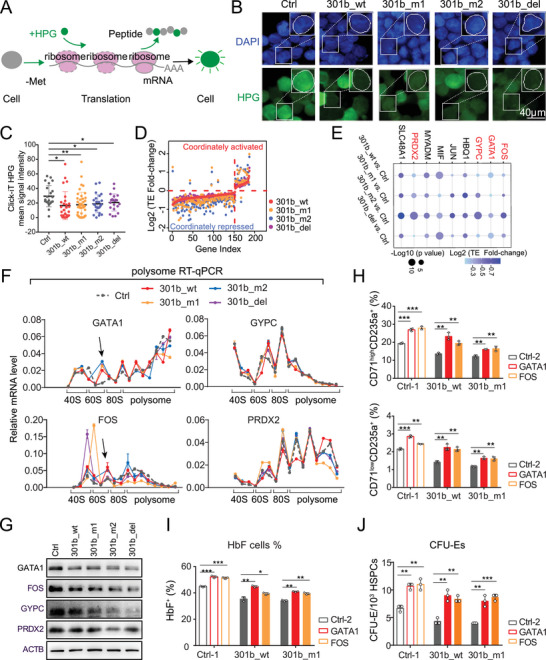
Molnc‐301b suppresses the translation of essential erythroid mRNAs. A) Experimental procedure for nascent protein synthesis detection (Click‐iT HPG mean signal intensity). B) Visualization of nascent protein synthesis in K562 cells treated with Ctrl, 301b_wt, 301b_m1, 301b_m2, and 301b_del. Scale bar, 40 µm. C) The yield of nascent proteins was measured by the amount of HPG incorporation in K562 cells treated with Ctrl, 301b_wt, 301b_m1, 301b_m2, and 301b_del. *p*‐Values were calculated using a two‐sided Mann‐Whitney test (*n* ≥ 20 cells for each). *p <0.05, **p <0.01. D) Scatterplots showing differential TE of 301b_wt‐, 301b_m1‐, 301b_m2‐, 301b_del‐overexpressing K562 cells compared with the control. Significantly TE up‐ or downregulated genes are determined by *p* <0.05 (*n* = 2 replicates). E) Plot showing the TE fold‐change of essential erythroid regulators. Circle size indicates ‐log10 (*p*‐Values) and circle color represents log2 (TE fold‐change). F) Polysome RT‐qPCR analysis of *GATA1, FOS, GYPC*, and *PRDX2* transcripts in K562 cells treated with Ctrl, 301b_wt, 301b_m1, 301b_m2 and 301b_del. 18S rRNA (Figure S6H, Supporting Information) was used for quality control (*n* = 3 replicates). G) Western blot validation of candidates in K562 cells treated with Ctrl, 301b_wt, 301b_m1, 301b_m2, and 301b_del. H‐I) Percentage of CD71^high^/CD235a^+^, CD71^low^/CD235a^+^ and HbF^+^ cells in Figure S7C, Supporting Information. Data represent the mean ± SD (*n* = 3 replicates). *p*‐Values were calculated by unpaired *t*‐test. *p < 0.05, **p <0.01 and ***p <0.001. J) Average frequency of colony formation per 10^3^ HSPCs. Data represent the mean ± SD (*n* = 3 replicates). *p*‐Values were calculated by unpaired *t*‐test. **p <0.01 and ***p <0.001.

Intriguingly, the expression of translation‐associated genes was significantly suppressed by molnc‐301b in the antagonistic molnc‐301b‐SMARCA5 axis. Thus, we speculated that molnc‐301b affects mRNA translation indirectly by regulating the expression of translation‐associated genes. To test the hypothesis, we performed polysome profiling in cells overexpressing molnc‐301b, which revealed a slight reduction in the polysome pools (Figure S6A, Supporting Information). In parallel, we tested the effects of molnc‐301b on global RNA translation by employing l‐homopropargylglycine (HPG), a methionine analog that is specifically incorporated in de novo protein synthesis, allowing the detection of nascent peptide synthesis by fluorescence (**Figure** **6**A). Compared with the control cells, HPG incorporation into nascent proteins was moderately reduced following molnc‐301b overexpression (Figure 6B,C). Op‐puro incorporation assay showed similar results (Figure S6B, Supporting Information). Additionally, the surface sensing of translation (SUnSET) assay^[^ ^29^ ^]^ also indicated a reduced protein production in molnc‐301b overexpressing cells (Figure S6C, Supporting Information). Therefore, these findings indicate that the overall protein translation is influenced to some extent by molnc‐301b.

To further determine the potential effects of molnc‐301b on the translation of individual genes, we performed Ribo‐seq^[^ ^30^ ^]^ analysis of K562 cells overexpressing wild‐type or mutant molnc‐301bs. The quality control results of Ribo‐seq (Figure S6E–G, Supporting Information, see Experimental Section) were consistent with previous studies.^[^ ^31^ ^]^ Calculation of the translation efficiency (TE) as the ratio of RPFs relative to mRNA abundance showed that TE was decreased for most mRNAs in molnc‐301b overexpressing cells (Figure 6D; Table S5, Supporting Information), thus indicating the translational suppression activity of molnc‐301b. Among these genes exhibiting downregulated TE, *GATA1*, *FOS*, *GYPC*, and *PRDX2* are essential hematopoietic regulators (Figure 6E). We verified the molnc‐301b‐mediated translation inhibition of GATA1 and FOS mRNAs via polysome profiling coupled with qPCR analysis (Figure 6F; Figure S6H,I, Supporting Information). Moreover, the translational repression activity of molnc‐301b ultimately resulted in decreased levels of GATA1 and FOS proteins (Figure 6G; Figure S6J–L, Supporting Information). Indeed, it has been observed that *GATA1* exhibits a shorter and more unstructured 5′ UTR than other transcripts, which might explain its translational sensitivity to reduced ribosome levels.^[^ ^27^ ^]^

Furthermore, we performed “rescue” assays by overexpressing GATA1 or FOS in molnc‐301b overexpressed cells (Figure S7A,B, Supporting Information). Flow cytometric analysis revealed that overexpression of either wild‐type or mutant molnc‐301bs decreased the percentage of differentiated erythroid cells (CD71^high^/CD235a^+^ and CD71^low^/CD235a^+^) and HbF^+^ cells. Subsequently, the re‐introduction of GATA1 or FOS restored the impaired erythroid differentiation caused by molnc‐301b overexpression (Figure 6H,I; Figure S7C, Supporting Information). CFU assays also showed a consistent result with flow cytometric analysis (Figure 6J; Figure S7D, Supporting Information). These results further verified that molnc‐301b attenuated erythroid differentiation by regulating the expression of GATA1 and FOS.

### 2.8 Establishment of a Functional molncRNA Screening Platform using CRISPR‐based Single‐Cell RNA‐seq (scRNA‐seq)

**Figure 7:**
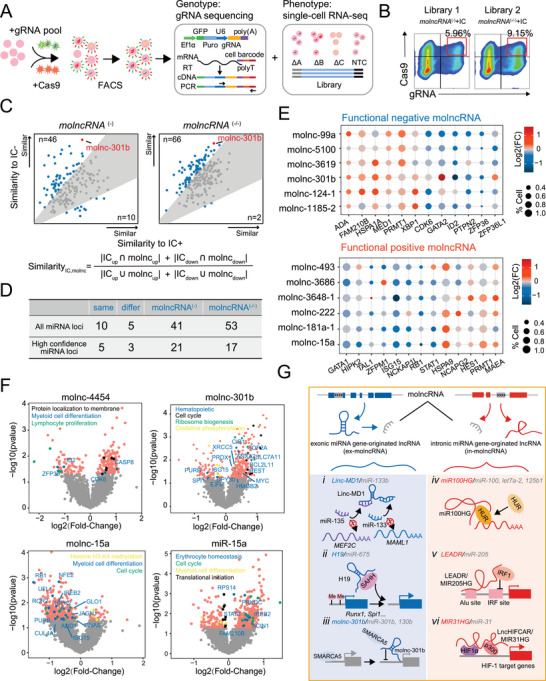
Functional molncRNA screening via CRISPR‐based scRNA‐seq. A) Schematic illustration of an integrated approach for CRISPR‐pooled screening of functional molncRNAs with single‐cell transcriptomics. B) Flow cytometric analysis of infected gRNA‐EGFP^+^/Cas9‐mCherry^+^ K562 cells. *MolncRNA* ^(‐)^ containing more than two pairs of gRNAs for each of the 361 miRNA loci targeted by D1 and D2 gRNAs recognizing the 1–3 kb or 0.5–1 kb downstream region of the miRNA precursor to specifically perturb molncRNA expression. *MolncRNA* ^(‐/‐)^ containing gRNAs for the same 361 target genes, but recognizing the 1–2 kb upstream or 1–3 kb downstream region of the *miRNA* precursors (U1 and D1 gRNAs) to knock out both molncRNAs and the cognate miRNAs. IC, internal control gRNA. C) Pairwise similarity of transcriptome changes between molncRNA and IC+/IC‐ genes. The similarity of transcriptome changes was calculated as the formula on the lower panel (the method referred to Tian's work^[^ ^77^ ^]^) D) Classification of functional molncRNA gRNAs by comparing with library 1 and library 2. E) Plot displaying a differential expression of erythroid‐associated genes in cells with the indicated functional molncRNA (upper panel, negative molncRNA regulators; lower panel, positive molncRNA regulators) compared to cells with NTC cells. Circle size represents the proportion of cells expressing indicated erythroid differentiation genes. F) Volcano plot showing the DEGs caused by molncRNA or miRNA knockdown. DEGs in red (*p*‐Value < 0.05), or other colors for genes belong to specific GO functional terms labeled in the diagram. G) *i*–*iii*. Models of ex‐molncRNA function. *i*. Linc‐MD1 binds miR‐133 and miR‐135 to act as a competing endogenous RNA (ceRNA) that abolishes miRNA repressing activity on MAML1 and MEF2C and controls muscle differentiation. *ii*. H19 binds to SAHH and inhibits its activity, thus mediating the demethylation of hematopoietic transcription factors (Runx1 and Spi1) in mouse embryonic hematopoiesis. *iii*. Molnc‐301b acts as an “RNA decoy” molecule by binding to SMARCA5 to perturb its interaction with chromatin. *iv*–*vi*) Models of in‐molncRNA function. *iv*) MIR100HG facilitates the interaction between HuR and its target mRNAs to regulate the cell cycle. *v*) LEADeR binds to promoters with an Alu element in the proximity of the interferon regulatory factor (IRF) binding site and negatively regulates prostate basal luminal differentiation. *vi*) LncHIFCAR/MIR31HG enhances the recruitment of HIF‐1α and p300 to HIF‐1 target promoters, and activates the HIF‐1 transcriptional network, with the ultimate effect of regulating cancer development.

To systematically identify functional molncRNAs in hematopoietic cells, we combined pooled CRISPR‐Cas9‐based screening with scRNA‐seq. In addition, we performed targeted amplification to more efficiently recover the guide RNAs (gRNAs) presented in each cell for better characterization of the genotype‐to‐phenotype relationships in single cells (**Figure** **7**A).^[^ ^32^, ^33^ ^]^

Here, we performed loss‐of‐function screening of 361 miRNA loci (Table S6, Supporting Information). First, to assess the function of molncRNAs, we introduced 12 internal control (IC) genes, consisting of six negative regulators (IC−: *GATA2*, *SPI1*, *ID2*, *SATB1*, *MAFB*, and *miR‐221/222*) and six positive regulators (IC+: *HMGB2*, *GATA1, NFE2, KLF1, STAT5A, MYB)* of erythroid differentiation. To distinguish the function of molncRNA and miRNA, we designed two pooled gRNA libraries. Library 1 (*molncRNA* ^(‐)^ and IC) contained more than two pairs of gRNAs for each of the 12 ICs and the 361 miRNA loci, which were targeted by D1 and D2 gRNAs recognizing the 1–3 kb or 0.5–1 kb downstream region of the miRNA precursors, to specifically perturb molncRNA expression (Figure S8A; Table S6, Supporting Information). Library 2 (*molncRNA* ^(‐/‐)^ and IC) contained gRNAs for the same target genes as Library 1 but recognized the 1–2 kb upstream or 1–3 kb downstream region of the miRNA precursors (U1 and D1 gRNAs) to knock out both the molncRNAs and their cognate miRNAs (Figure S8A; Table S6, Supporting Information). Cells without gRNA were used as the non‐targeting control (NTC).

Next, these libraries were cloned into lentiviral vectors (pLV‐hEF1a‐EGFP‐2A‐Puro‐U6‐gRNA1‐7SK‐gRNA2) and modified using the CRISPR droplet sequencing (CROP‐seq) method.^[^ ^34^ ^]^ Two tandem‐double‐gRNA‐plasmid libraries with EGFP green fluorescence were obtained. We then infected K562 cells with a mix of lentiviruses expressing Cas9‐mCherry (approximate multiplicity of infection (MOI) 50) and gRNA‐EGFP (Library 1 and 2; approximate MOI 4) (Figure S8B, Supporting Information). Infected EGFP^+^/mCherry^+^ cells were sorted for scRNA‐seq using the 10× Genomics scRNA‐seq poly(A)‐primed platform, and their corresponding gRNAs were further amplified specifically using a previously described protocol^[^ ^33^ ^]^ (Figure 7A,B).

The subsequent analysis was based on 56509 high‐quality single‐cell transcriptome profiles with unique gRNA assignments and an average of ≈100 cells per gene targeted (Figure S8C–E; Table S6, Supporting Information). Of note, most gRNA‐targeted cells (79%–84%) were assigned to one gRNA, except for a small fraction of cell doublets that matched more than one gRNA (Figure S8D, Supporting Information). After scRNA‐seq quality control (Figure S8F, Supporting Information), 3848 NTC cells and 8729 cells containing IC gRNAs were used as references.

We also compared the transcriptomes of different gRNAs targeting the same internal control genes. The multiple gRNA groups targeting the same gene tended to cluster together, confirming that independent gRNAs targeting the same gene had similar phenotypic consequences (Figure S8G, Supporting Information). In the molncRNA^(‐)^ and molncRNA^(‐/‐)^ libraries, ≈44–46% of molncRNA gRNAs clustered closely with each other targeting the same molncRNA. To detect the reliable transcriptome perturbation after IC gene knockdown, we selected those cells in which the targeted IC was successfully repressed for further analysis (Figure S8H, Supporting Information).

### 2.9 Identification of Functional molncRNAs

Next, we performed uniform manifold approximation and projection (UMAP) of gRNAs. In the molncRNA^(‐/‐)^ and molncRNA^(‐)^ libraries, ten distinct clusters were identified (Figure S8I, Supporting Information, left panel). Nevertheless, cell clusters did not match gRNA groups (Figure S8I, Supporting Information, right panel). GO functional enrichment analysis showed that the top 50 genes in the first and second principal components (PC1 and PC2) were involved in cell cycle‐associated pathways, which is a reflection of differences in cell states (Figure S8J, K, Supporting Information). Therefore, the UMAP analysis primarily reflects the difference between cell states rather than molecular phenotypes caused by gRNA. Then to examine the transcriptome changes of gRNA, we performed differential expression analysis between molncRNAs‐ or IC ‐targeted cells and the NTC cells. We found that the knockdown of IC genes did cause expression changes of hematopoietic‐related genes (Figure S8L, Supporting Information). Subsequently, we merged the DEGs in IC+ and IC‐ groups separately and removed DEGs overlapped in both groups, which might be a reflection of the phenotype triggered by gRNA infection. The filtered DEGs could separate cells with IC+ or IC‐ gRNAs (Figure S8M, Supporting Information).

Next, we determined the functional molncRNAs by comparison of similarities in transcriptome changes between molncRNAs‐ and IC‐targeted cells (Figure 7C). The positive molncRNAs (Figure 7C) were defined if their transcriptome changes had more similarities to positive erythroid regulators (IC+), while the negative molncRNAs (Figure 7C) were that had more similarities to negative regulators (IC‐, see Experimental Section). Based on this, we identified 124 functional molncRNA gRNAs (112 negative and 12 positive gRNAs) in erythropoiesis. Among them, 46 molncRNA gRNAs target high‐confidence miRNA loci (see Experimental Section). To distinguish the function of lncRNA or miRNA, molncRNAs were divided into three categories by comparison of phenotypes of libraries 1 and 2: *i)*. same, lncRNA and miRNA might have similar phenotypes; *ii)* different, lncRNA and miRNA have opposite phenotypes; *iii)* molncRNA^(‐)^ only, lncRNA has functions independent of its cognate miRNA (Figure 7D). Finally, we screened out 29 functional molncRNAs (in “same”, “different”, “molncRNA^(‐)^ only” group). Accordingly, cells expressing negative molncRNAs gRNAs showed increased expression of erythroid positive regulators (e.g., *MED1 and PRMT1*), but decreased expression of negative regulators (e.g., *GATA2 and ID2*) (Figure 7E, upper panel). In contrast, in most cases, the opposite expression pattern of erythroid regulators was observed in cells expressing positive molncRNAs gRNAs (Figure 7E, lower panel).

Among the 29 functional molncRNAs, 21 were only detected in the *molncRNA* ^(‐)^ library (“molncRNA only” group), such as molnc‐4454, affecting the myeloid cell differentiation and lymphocyte proliferation gene expression (Figure 7F); 5 (“same” group) have similar function with their cognate miRNAs, such as molnc‐301b, supportive of the accuracy of screening results. Moreover, CROP‐seq also showed that molnc‐301b knockdown could down‐regulate the expression of hematopoietic differentiation or translational‐related genes (Figure 7F). Additionally, 3 molncRNAs (“different” group) were shown to have opposite functions to their cognate miRNAs, such as molnc‐15a/DLEU2. Molnc‐15a/DLEU2 is predicted to promote erythroid differentiation and affect myeloid cell differentiation‐ and cell cycle‐related gene expression (Figure 7F). Meanwhile, miR‐15a knockdown upregulated the expression of cell cycle‐related genes and inhibited erythroid differentiation, which is consistent with previous studies^[^ ^35^ ^]^ (Figure 7F). Notably, 17 functional molncRNAs were also identified by PacBio Iso‐seq in Figure 1. The negative regulators molnc‐1206 and −3648‐1 were downregulated at days 14 and 18 during erythroid differentiation, providing further evidence of their roles as functional lncRNAs.

## 3 Conclusion

More than 70% of miRNA genes in mammals are located in introns.^[^ ^36^ ^]^ When miRNAs are derived from the introns of non‐coding transcripts, host genes may be considered as only pri‐miRNAs, non‐functional by‐products of miRNA processing, or lncRNAs with independent functions.^[^ ^14^ ^]^ This makes us speculate that, apart from participating in miRNA processing, other mechanisms of action of pri‐miRNA remain to be discovered. Here, we conducted systematic screening and identification of miRNA gene‐originated lncRNAs expressed in hematopoietic cells based on single‐molecule long‐read sequencing technology.

Using PacBio Iso‐seq combined with RNA‐seq, we found that molncRNAs had long half‐lives and showed dynamic expression during hematopoietic differentiation. Among them, molnc‐301b functions as a “decoy” of SMARCA5 to suppress the expression of erythropoiesis‐ and translation‐associated genes at the transcriptional level. In addition, we showed that molnc‐301b also impeded the translational process of several erythroid gene‐derived RNAs, and thereby attenuated erythropoiesis at post‐transcriptional level. Finally, we developed a CRISPR‐based platform for molncRNAs screening at single‐cell resolution and eventually identified 29 functional molncRNAs in hematopoietic cells. Such a large‐scale screening system is also applicable to identify content‐specific and function‐essential molncRNAs in other tissues and disease models.

Based on whether miRNAs were located in the exonic or intronic region of their host genes, molncRNAs were divided into exonic miRNA gene‐originated lncRNA (ex‐molncRNA) and intronic miRNA gene‐originated lncRNA (in‐molncRNA). Accordingly, examples of both exonic and intronic miRNA gene‐originated lncRNAs have been reported in previous studies. Among them, several ex‐molncRNAs had been functionally investigated before they were identified as pri‐miRNAs (Figure 7G), such as lncRNA‐H19 from miR‐675 locus. H19 maintains HSC quiescence in the adult bone marrow by restricting IGF2‐IGF1R signaling in a miRNA‐dependent manner.^[^ ^11^ ^]^ Nevertheless, during embryonic hematopoiesis, H19 promotes pre‐HSC and HSC specifications by regulating the demethylation of hematopoietic transcription factors (e.g., *Runx1* and *Spi1*) in a miR‐675‐independent manner.^[^ ^12^ ^]^ These findings suggest the functional diversity of H19 in regulating embryonic emergence versus adult homeostasis of HSCs. Furthermore, whether the dominant function of different phenotypes is mediated by lncRNAs or miRNAs, requires further investigation. Another muscle‐specific lncRNA, linc‐MD1, is transcribed by a genomic locus containing *miR‐206* and *miR‐133b*. Linc‐MD1 “sponges” miR‐133 and miR‐135 and abolishes miRNA repression of MAML1 and MEF2C.^[^ ^13^ ^]^ Additionally, in‐molncRNAs are also biologically relevant in different contexts (Figure 7G). For instance, miR‐100, miR‐let7a‐2, and miR‐125b1 are embedded in the intron of the *MIR100* host gene (*MIR100HG*), and *MIR100HG*‐encoded lncRNA regulates the cell cycle by interacting with HuR to influence its association with target mRNAs.^[^ ^37^ ^]^ Similarly, *pre‐miR‐205* is located in the last intron/exon junction of *MIR205HG*. The processed transcript serves as a lncRNA that operates autonomously from miR‐205 in prostate basal cell differentiation and has been reannotated as long epithelial Alu‐interacting differentiation‐related RNA (LEADeR).^[^ ^14^ ^]^ MiR‐31 is harbored in the first intron of *MIR31HG*, which was identified as a HIF‐1α co‐activating lncRNA and renamed LncHIFCAR. LncHIFCAR enhances HIF‐1 complex binding to the target loci and facilitates the recruitment of p300, driving oral cancer progression.^[^ ^38^ ^]^

It became evident that the repertoire of genome‐encoded RNAs is far more extensive and complex than previously thought. Daniel He et al. also found four miRNA gene‐originated lncRNAs that regulate cell proliferation even when miRNA production machinery is knocked down.^[^ ^17^ ^]^ The finding of molncRNAs gives insight into bifunctional loci, one locus with two roles. Likewise, different functions might be assigned to molncRNAs and the derived miRNAs in different biological processes. For example, our CRISPR‐based screen revealed that molnc‐301b and miR‐301b act synergistically to inhibit erythroid differentiation, whereas molnc‐15a and miR‐15a exhibit antagonistic functions in erythropoiesis. Thus, the pervasive transcription of complex genomes provides ample material for the functional innovation of diverse lncRNAs.

In summary, taking advantage of state‐of‐art technologies, including PacBio Iso‐seq, CRISPR screen, and scRNA‐seq, we have characterized over 860 miRNA gene‐originated lncRNAs, which expanded our understanding of the transcripts overlapped with miRNA loci, as previous studies reported lnc‐pri‐miRNAs or miRNA host gene‐encoded transcripts. Besides, we clarified their essential biological functions during hematopoiesis and uncovered the underlying mechanisms by which molncRNAs steer target gene expression at both transcriptional and post‐transcriptional levels. Our study suggests a previously unappreciated and new potential regulatory role of miRNA host genes, the functions of which can be mediated by either miRNAs or the derived molncRNAs.

## 4 Experimental Section

### Human HSPCs

Human umbilical cord blood was obtained from normal full‐term deliveries after informed consent as approved by the Research Ethics Committee of the Institute of Basic Medical Sciences, Chinese Academy of Medical Sciences (project number, 2019015). After isolation by HISTOPAQUE (Sigma‐Aldrich, Deisenhofen, Germany) density gradient centrifugation, CD34^+^ cells were enriched from Mononuclear cells through positive immunomagnetic selection (CD34 MultiSort kit, Miltenyi Biotec, Bergisch‐Glad‐bach, Germany). The isolated CD34^+^ HSPCs were differentiated ex vivo toward the erythroid lineage. HSPCs were cultured in IMDM supplemented with serum substitute (BIT; Stem Cell Technologies, Vancouver, BC, Canada), 2 mM l‐Glutamine (Gibco), 40 µg mL^−1^ inositol (Sigma), 10 µg mL^−1^ folic acid (Sigma), 90 ng mL^−1^ Ferrous nitrate (Sigma), 900 ng mL^−1^ Ferrous sulfate (Sigma), 1.6×10^−4^ m Monothioglycerol (Sigma). From days 0 to 8, 3 ng mL^−1^ recombinant human EPO (PeproTech, Rocky Hill, NJ, USA), 100 ng mL^−1^ recombinant human stem cell factor SCF (PeproTech), 5 ng mL^−1^ recombinant human IL‐3 (PeproTech), 10^−6^ m Hydrocortisone (Sigma). From days 8 through 14, SCF and EPO were included in the culture medium, and only EPO was included during days 14 to 18. Cells were harvested every 3–5 days.

### hESC Cells

The H1 hESC line (NIH code WA01), obtained from the WiCell Research Institute, was maintained on matrigel (Corning, New York, USA)‐coated dishes (6‐well plate) in mTeSR1 medium (Stem Cell Technologies). The fresh medium was changed every day.

### CRISPR/Cas9 Mediated Knockin in hESC

For knockin, the targeting vector contains two homology arms (≈1500 bp in length) and an expression cassette of puromycin‐resistant gene flanked by two loxP sites, followed by the 3×SV40 poly(A) signal sequence and a BGH poly(A) signal (a total of 4×poly(A) stop signal). It was worth noting that, to eliminate the confusion of mature miR‐301b, a miR‐301b precursor sequence upstream of the 4×poly(A) was also incorporated to maintain miR‐301b expression upon molnc‐301b termination. The targeting vectors were co‐electroporated into H1 hESCs with plasmids expressing Cas9 and two gRNAs. After 48 hours of puromycin‐selection, hESC colonies were picked, expanded, and analyzed to identify insertions. The gRNAs and primers are listed in Table S7 (Supporting Information).

### hESC‐based Erythroid Differentiation

Hematopoietic differentiation of hESCs was carried out as previously described.^[^ ^24^ ^]^ In brief, hESCs were dissected into single cells using Accutase (Gibco) and plated on growth factors reduced (GFR) Matrigel‐coated dishes at a density of 3 × 10^4^ cells per well (12‐well plate) in mTeSR1 medium containing 5 µM Y27632 (MedChemExpress, New Jersey, USA). 24 h later, cells were replaced with Custom mTeSR1 medium (Stem Cell Technologies) supplemented with 50 ng mL^−1^ Activin A (Peprotech) and 40 ng mL^−1^ BMP4 (Peprotech) for 2 days. From days 3 to 4, cells were incubated with Custom mTeSR1 medium supplemented with 40 ng mL^−1^ VEGF (Peprotech) and 50 ng mL^−1^ bFGF (Peprotech). From days 5 to 7, cells were incubated with Custom mTeSR1 medium supplemented with 40 ng mL^−1^ VEGF, 50 ng mL^−1^ bFGF, and 20 µM SB431542 (Stem Cell Technologies) for 3 days. Then, we could observe cobble‐like hematopoietic regions forming cells. Next, cells were dissociated and the differentiation efficiency was determined by flow cytometry. Single cells were seeded into low‐attachment 6‐well plate and cultured for 6 days in Custom mTeSR1 medium containing 20 ng mL^−1^ SCF (Peprotech), 50 ng mL^−1^ TPO (Peprotech), 20 ng mL^−1^ IL‐3 (Peprotech), 1 mM L‐Glutamine (Gibco), 2% B27 (Gibco), 0.1 mM MTG (Sigma‐Aldrich), 1% ITS (Gibco), 1% NEAA (Gibco). The fresh medium was changed every 2 days and the differentiation efficiency was determined on day 4/6 by flow cytometry. CD43^+^ cells were selected and subjected to colony‐forming unit assay.

### Cell Lines and Culture Conditions

Human erythroleukemia cell line K562 was maintained in RPMI 1640 medium supplemented with 10% FBS (Gibco, Carlsbad, CA, USA). 293T cells were obtained from the cell resource center of the Institutes of Basic Medical Sciences, Chinese Academy of Medical Sciences, and grown in DMEM with 10% FBS.

### RNA Extraction and Quantitative Real‐Time PCR (qRT‐PCR)

Total RNA was extracted using Trizol (Invitrogen, Carlsbad, CA, USA), and cDNA was synthesized using M‐MLV reverse transcriptase (Promega, Madison, WI, USA) from 1–4 µg of total RNA. qRT‐PCR was carried out in the Bio‐Rad CFX‐96 (Bio‐Rad, Foster City, CA, USA) using SYBR Premix Ex Taq kit (Takara, Dalian, China). Each assay was performed in triplicate. Primer sequences used for qRT‐PCR are shown in Table S7 (Supporting Information).

### Flow Cytometry

CD34^+^ HSPCs were harvested at indicated times and washed twice at 4 °C in PBS. Approximately 1×10^5^ cells were washed and resuspended in PBS and stained with PE‐conjugated anti‐CD235a and APC‐conjugated anti‐CD71 (eBioscience, CA, USA) at 4 °C for 30 min. After the incubation, cells were washed with PBS, resuspended in 4% PFA (Solarbio, Beijing, China), and subjected to flow cytometric analysis. For HbF analysis, HSPCs were washed with PBS and resuspended in 4% PFA at room temperature (RT) for 10 min. Cells were then washed with PBS, and resuspended in PBS/0.1% Triton X‐100 at RT for 10 min. Next, cells were washed and resuspended in PBS and stained with PE‐conjugated anti‐HbF at 4 °C for 1 h. Flow cytometry was carried out on a C6 Flow Cytometer Instrument (BD Biosciences, San Jose, CA, USA). hESCs were collected on the fourth day of suspension culture and stained with PE‐conjugated anti‐CD34 and PE‐conjugated anti‐CD45, respectively. On the fourth day of erythroid differentiation, hESCs were collected and stained with PE‐conjugated anti‐CD235a and APC‐conjugated anti‐CD71 at 4°C for 30 min.

### RNA Stability Assay and Sequencing for molncRNA Lifetime

K562 cells were treated with 1 µg mL^−1^ actinomycin D and collected at indicated time points. Total RNA was extracted using Trizol (Invitrogen). For RNA sequencing, an equal amount of ERCC RNA spike‐in control (Thermo Scientific, Waltham, MA) was added to the total RNA samples as internal control before library construction. Sequencing libraries were prepared using NEBNext Ultra Directional RNA Library Prep Kit. RNA stability assay was generated from two biological replicates.

### RACE Analysis

To isolate the full‐length molnc‐301b, 5′ and 3′ RACE reactions were performed on poly(A)^+^ RNA of K562 cells using the SMARTer RACE 5′/3′ Kit (TaKaRa, Dalian, China) according to the manufacturer's protocol. Primers used for the RACE experiment were listed in Supporting Information, Table S7.

### Plasmid Construction

For molnc‐301b overexpression, a 2460 bp 301b_wt) was amplified from genomic DNA of K562 cells and cloned into a pWPXL vector. The miRNA seed sequence mutant/delete molnc‐301b constructs (301b_m1, 301b_m2, and 301b_del) were created using the QuickChange Site‐Directed Mutagenesis kit (Agilent Technologies, La Jolla, CA, USA). For miRNAs overexpression, pre‐miR‐301b/130b was inserted into pWPXL vector. The primers are listed in Table S7 (Supporting Information).

### Lentivirus Production and Cell Infection

Recombination lentiviruses for molnc‐301b and miR‐301b/130b overexpression were produced using a pWPXL vector. For lentivirus production, lentiviral vectors were co‐transfected into 293T cells with packaging vectors psPAX2 (#12260, Addgene) and pMD2.G (#12259, Addgene) using lipofectamine LTX (Invitrogen, USA). Infectious lentivirus particles were harvested at 48 h after transfection, and filtered through 0.45 µm PVDF filters. The harvested viral particles were added into the HSPCs plus with 8 µg mL^−1^ polybrene for 12 h. Then the cells were replaced with fresh complete medium and subjected to the following experiments. 2 unique shRNA constructs in lentiviral GFP vector of SMARCA5 (TL309248, ORIGENE) were used to package lentivirus and infect K562 cells. After 48 h of infection, the cells were selected with 1 µg mL^−1^ puromycin until the end of the culture. For RNA‐seq, differentiated HSPCs at days 11, 14, and 18 and infected K562 cells were collected, and poly(A)^+^ RNA was enriched and sequenced at Novogene (Tianjin, China).

### Colony‐Forming Unit Assay

Lentivirus‐infected HSPCs or CD43^+^ hESCs were plated in a 35 mm Petri dish containing 2 mL methylcellulose medium (Stem Cell Technologies, Vancouver, BC, Canada) supplemented with rh SCF, rh GM‐CSF, rh IL‐3, rh IL‐6, rh G‐CSF, rh EPO. The cells were incubated at 37 °C with 5% CO_2_ for 10 days for CFU‐E quantification.

### Subcellular Fractionation

Cells (6 × 10^6)^ were washed in PBS and suspended in 400 µL Solution A (10 mM HEPES, 10 mM KCl, 1.5 mM MgCl_2_, 0.34 m sucrose, 10% glycerol, 1 mM DTT, 1×protease inhibitor cocktail (Roche Life Science, Indianapolis, USA), 0.1% Triton X‐100). Mixed gently and incubated on ice for 5 min. The cytoplasmic and nuclear fractions were harvested by centrifugation at 1300 g for 4 min. The isolated nuclei were washed in 1 mL Solution A and incubated with 400 µL Solution B (3 mM EDTA, 0.2 mM EGTA, 1 mM DTT, protease inhibitor cocktail) on ice for 30 min. The nucleoplasm and chromatin fractions were separated by centrifugation at 1700 g for 4 min.

### RNA Pull‐Down

molnc‐301b RNA was transcribed in vitro using RiboMAX large‐scale RNA production systems‐T7 (Promega). A single biotinylated nucleotide was added to the 3′ terminus of an RNA strand with the Pierce RNA 3′ End Desthiobiotinylation Kit (Thermo Scientific). RNA Pull‐down assay was performed with the Pierce Magnetic RNA‐protein Pull‐Down Kit (Thermo Scientific) according to the manufacturer's instruction. In brief, 50 pmol of biotinylated RNA was mixed with 50 µL washed Streptavidin agarose beads (Invitrogen) and incubated at room temperature with agitation for 30 min. 2 × 10^7^ K562 cells were lysed in standard lysis buffers (25 mM Tris‐HCl pH 7.4, 150 mM NaCl, 1% Np‐40, 1 mM EDTA, 5% glycerol) supplemented with protease inhibitor cocktail and 1 U µL^−1^ RNase Inhibitor on ice for 30 min followed by centrifugation. Then the RNA‐binding beads were added to the cell lysate and rotated at 4 °C for 1 h. Beads were washed with 20 mM Tris‐Cl three times and boiled in 1×SDS loading buffer. The proteins were detected by Western blot or separated in gradient gel electrophoresis followed by MS identification.

### Western Blotting and Silver Staining

Protein samples were separated on 10% SDS‐PAGE gels and then transferred to PVDF membranes. Membranes were blocked with 5% skimmed milk in TBS‐T and incubated with primary antibodies at 4 °C overnight. Membranes were washed and incubated with HRP‐conjugated secondary antibodies for 1 h at room temperature. Finally, membranes were washed and visualized with ECL substrate (Millipore).

Silver staining was performed according to the instructions. Briefly, gels were fixed with 40% ethanol: 10% acetic acid solution for 30 min, sensitized with 0.02% Na_2_S_2_O_3_ for 30 min, stained with 0.2% AgNO_3_: 0.02% formaldehyde for 20 min, and developed with 3% Na_2_CO_3_: 0.01% formaldehyde. The developing reaction was stopped using 1.46% EDTA‐2Na•2H_2_O.

### RNA Immunoprecipitation

To enable RNA Immunoprecipitation, cells were washed twice with PBS, collected and then the pellet was resuspended in IP lysis buffer (20 mM HEPES pH 7.9, 150 mM NaCl, 0.5 mM EDTA pH 8.0, 10 mM KCl, 1.5 m MgCl_2_, 0.5% Np‐40, 10% glycerine, 1.5 mM DTT, protease inhibitor cocktail, 10 U mL^−1^ RNase Inhibitor) and then left on ice for 30 min followed by centrifugation. The supernatant was transferred to tubes with SMARCA5 antibody (Abcam, ab3749) and 40 µL of protein G beads (Invitrogen), and IP was performed by tube rotating at 4 °C overnight. After washed three times with wash buffer (20 mM HEPES pH 7.9, 150 mM NaCl, 0.5 mM EDTA pH 8.0, 10 mM KCl, 1.5 M MgCl_2_, 0.5% Np‐40, 10% glycerine, 1.5 mM DTT, 1 mM PMSF), co‐precipitated RNA was extracted by Trizol reagent.

### Chromatin Isolation by RNA Purification (ChIRP)

The ChIRP procedure was performed as described previously with the following modifications.^[^ ^39^ ^]^ DNA probes were 22 nt and biotinylated (Tianyi Biotech). molnc‐301b ChIRP probes were listed in Table S7 (Supporting Information).

About 2 × 10^7^ K562 were harvested and washed twice by ice‐cold PBS. Cells were then cross‐linked by 1% glutaraldehyde at room temperature for 10 min, followed by quenching the cross‐linking reaction with 1/10th volume of 1.25 m glycine at room temperature for 5 min. The cells were then washed twice by ice‐cold PBS. Snap frozen by liquid nitrogen and stored at −80 °C.

Crosslinked cell pellets were resuspended in 1 mL nuclei lysis buffer (50 mM Tris‐Cl pH 7.0, 10 mM EDTA, 1% SDS) with the addition of a protease inhibitor cocktail., PMSF and RNase Inhibitor. Chromatin fractions were sonicated to have average DNA size ranging from 100–500 bp and spun at 4 °C at 16 100 g for 10 min. Cleared chromatin supernatant was saved for probe hybridization. For a typical ChIRP sample using 1 mL of lysate, remove 10 µL for RNA INPUT and 10 µL for DNA INPUT and place in Eppendorf tubes. Keep on ice till further use. Add 2 mL Hybridization Buffer (750 mM NaCl, 1% SDS, 50 mM Tris‐Cl pH 7.0, 1 mM EDTA, 15% formamide, protease inhibitor cocktail, PMSF, RNase Inhibitor) and 100 µM probes to 1 mL of lysate. Mix well. Incubate at 37 °C with shaking overnight. Then, 100 µL of T‐1 magnetic beads were added and incubated at 37 °C for 30 min with shaking. The beads were then washed 5 times at 37 °C with 1 mL wash buffer (2× SSC, 0.5% SDS, PMSF). 5 min of end‐to‐end rotation per wash was allowed. At the last wash, resuspend the beads well. Remove 100 µL and set aside for RNA isolation. Reserve 900 µL for DNA fraction.

For RNA isolation, 100 µL beads were resuspended in 95 µL RNA PK Buffer (100 mM NaCl, 10 mM TrisCl pH 7.0, 1 mM EDTA, 0.5% SDS, 1 µg µL^−1^ protease K) and incubated at 50 °C for 45 min with end‐to‐end shaking, including RNA INPUT. Then the beads were boiled at 95 °C for 10 min. RNA was purified by Trizol reagent.

For DNA isolation, the beads were further washed twice with DNA elution buffer (50 mM NaHCO_3_, 1% SDS, 100 µg mL^−1^ RNase A and 100 U mL^−1^ RNase H) at 37 °C for 30 min, including DNA INPUT. Then the crosslinking was reversed in the presence of 1 µg µL^−1^ protease K at 50 °C for 45 min. DNA was purified by PhOH:Chloroform: Isoamyl. Libraries were produced by employing the NEBNext Ultra RNA Library Prep Kit for Illumina (New England BioLabs).

### Chromatin Immunoprecipitation and qPCR (ChIP‐qPCR)

K562 cells (1 × 10^7)^ per sample were cross‐linked with 1% formaldehyde for 15 min. Cross‐linking was neutralized with 0.125 m glycine, and cells were rinsed in PBS twice. Then chromatin was sonicated using a Diagenode Bioruptor (Diagenode, Seraing, Belgium) for 30 min with 30 s pulse/pause cycles in polycarbonate tubes on ice to break chromatin into 200–500 bp fragments. Unbroken debris was spun down, and then the chromatin was split into two equal portions. One was used for the control IgG antibody (Millipore, Darmstadt, Germany), and the other portion was incubated with SMARCA5 antibody (Abcam, ab3749). Salmon sperm‐coated protein A/G beads (Millipore) were added to the two portions of the chromatin with equal volume. Then, the mixture of chromatin‐antibody‐protein‐A/G beads was incubated overnight at 4 °C. After washing four times, immunoprecipitated DNA was eluted from beads and purified for subsequent qPCR test. All the ChIP‐qPCR primers used in this study are listed in Supporting Information, Table S7 (Supporting Information).

### DNA EMSA

The biotin‐labeled target DNA probes, as well as corresponding cold probes, were synthesized by Tianyibiotech company (Tianyibiotech, Beijing, China). A total of 20 fmol of biotin‐labeled DNA probes were incubated with 1 ug SMARCA5 recombinant protein purchased from Origene (Origene Technologies, Rockville, MD, USA) using the LightShift Chemiluminescent EMSA Kit Pierce, IL, USA) according to the manufacturer's protocol.

Competition experiments were performed with a 200‐fold molar excess of the unlabeled probes (cold probe) or 7/14 ug molnc‐301b RNA preincubation. The reactions were incubated at room temperature for 20 min before adding DNA loading dye and separated by native 8% PAGE. The probes used for the DNA EMSA experiment are listed in Table S7 (Supporting Information).

### SunSET

For SUnSET assays,^[^ ^29^ ^]^ K562 cells treated with ctrl or molnc‐301bs were seeded at 4 × 10^5^ cells mL^−1^ in 6‐well plates. The control group was treated with 100 µg mL^−1^ of cycloheximide (CHX, Sigma) and puromycin. Puromycin pulses were performed by incubating the cells with 15 uL of 10 µg mL^−1^ puromycin for 15 min at 37 °C. Cells were then washed with cold PBS and lysed in RIPA buffer supplemented with 1 mM PMSF and protease inhibitor mixture. 5–10 µg of the whole cell lysate were assayed by Western blot analysis using the anti‐puromycin antibody.

### Measurement of Protein Synthesis

HPG/OPP incorporation assays were performed to detect nascent protein synthesis using Click‐iT HPG Alexa Fluor Protein Synthesis Assay Kits and Click‐iT Plus OPP Protein Synthesis Assay Kits (Life Technologies). HPG or OP‐Puro were added to the culture medium for 1 h, then cells were removed from wells and washed twice with PBS. Cells were fixed in 0.5 mL of 1% paraformaldehyde in PBS for 15 min on ice. Cells were washed in PBS, then permeabilized in 200 µL PBS supplemented with 0.5% Tx‐100 (Sigma) for 20 min at room temperature. The azide‐alkyne cycloaddition was performed using the Click‐iT Cell Reaction Buffer Kit and azide conjugated to Alexa Fluor 488 (Life Technologies) at 5 µM final concentration. After the 30‐min reaction, the cells were washed twice in PBS supplemented with 3% fetal bovine serum and 0.1% saponin, then resuspended in PBS supplemented with 4′,6‐diamidino‐2‐phenylindole (DAPI; 4 µg mL^−1^ final concentration) and analyzed by flow cytometry.

### Polysome Profiling

Cells were incubated with 100 µg mL^−1^ CHX (Sigma) at 37°C for 10 min, washed twice with ice‐cold PBS containing 100 µg mL^−1^ of CHX, and lysed in lysis buffer (100 mM KCl, 0.1% Triton X‐100, 50 mM HEPES, 2 mM MgCl_2_, 10% glycerine, 1 mM DTT, 100 µg mL^−1^ CHX, 20 U mL^−1^ RNase Inhibitor, protease inhibitor cocktail.). The lysate was collected, loaded onto a 10%−50% linear sucrose gradient containing 10 mM Tris‐HCl, 5 mM MgCl_2_, 100 mM NaCl, 1 mM DTT, and then centrifuged at 4 °C for 4 h at 27 500 rpm (Beckman, rotor SW28). The sample was then fractioned and analyzed by a Gradient Station (BioCamp) equipped with an ECONO UV monitor (BioRad) as well as a fraction collector (FC203B, Gilson). RNA was purified by Trizol from each fraction and subjected to RT‐qPCR analysis.

### Ribosome Profiling

The ribosome profiling procedure was performed as described previously with the following modifications (*n* = 2 biological replicates).^[^ ^40^ ^]^ Briefly, 500 µL lysate was prepared as described under polysome profiling. 100 µL lysate was used to prepare the total RNA library, meanwhile, ribosome footprinting and subsequent library preparation of ribosome‐protected RNA fragments (RPFs) was performed with 400 µL lysate. Add 10 U RNase I to 400 µL lysate and incubate for 45 min at room temperature with gentle mixing. Then the nuclease digestion was stopped by adding 1 µL RNase inhibitor. RPFs were purified with MicroSpinS‐400 columns (GE Healthcare Life Sciences) followed by size selection, which was conducted using 15% TBE‐urea gel. rRNAs were depleted with the NEBNext rRNA depletion kit (New England BioLabs). Following end repair and 3′adaptor ligation, RNAs were reverse transcribed using SuperScript III (Thermo Fisher, USA).

### CRISPR Screen

The single gRNAs were designed according to the *pre‐miRNA* sequences based on the publicly available online tool (http://crispr.mit.edu/). Upstream 1 (U1) gRNAs were designed within the range of 1–2 kb upstream of the target miRNA precursors; Downstream 1 (D1) gRNAs were designed within the range of 1–3 kb downstream of the target miRNA precursors; Downstream 2 (D2) gRNAs were designed within the range of 0.5–1 kb downstream of the target miRNA precursors. In addition, we selected 12 positive/negative regulatory genes in the erythroid differentiation as IC+ or IC−. Each gene was designed with ≥2 pairs of gRNAs. The gRNAs are listed in Table S6 (Supporting Information).

The library construction strategy was introduced as follows: Library 1 was designed to knock out the 3′end of molncRNAs and retain the expression of the corresponding miRNA. Library 1 (*molncRNA* ^(‐)^ and IC) included D1 and D2 gRNAs and also contained 102 pairs of IC gRNAs. Library 2 was designed to knock out the molncRNA and its cognate miRNA at the same time. Library 2 (*molncRNA* ^(‐/‐)^ and IC) included U1 and D1 gRNAs and IC gRNAs. Finally, these gRNAs were connected to the tandem double gRNA expression plasmid (pLV‐hEF1a‐EGFP‐2A‐Puro‐U6‐gRNA1‐7sK‐gRNA2) to obtain the gRNA libraries expressing EGFP green fluorescence.

To sort red+ and green+ populations, 3 × 10^6^ of K562 cells in 7.5 mL media plus 8 µg mL^−1^ polybrene were transduced, with either of the EGFP (MOI ≈4) or mCherry (MOI ≈50) expressing viruses that had been packaged singly. Infected cells were collected and subjected to scRNA‐seq. The libraries were prepared using a Single Cell 3′ Library Gel Bead kit V2 in replicate 1 and Single Cell 3′ Library Gel Bead kit V3 in replicates 2, 3 following the manufacturer's instructions.

### Specific Amplification of Guide Barcodes

The enriched PCR procedure was performed as described previously^[^ ^33^ ^]^ with the following modifications. A heminested PCR starting from 40 ng of full‐length cDNA was used to enrich for gRNA barcodes. Q5 High‐Fidelity DNA Polymerase (New England BioLabs) was used for PCR amplification according to the following PCR protocol: 1) 98 °C for 45 s, 2) 98 °C for 15 s, 65 °C for 20 s, then 72 °C for 60 s (14‐16 cycles). PCRs were cleaned with Zymo DNA Clean & Concentrator‐5 columns (Zymo, Orange County, CA, USA) and 15 µL of a 1:2 dilution of the first PCR and a 1:2 dilution of the second PCR were carried in each reaction. Then fragments of length 400–500 bp were selected using 8% TBE gel. Guide barcode libraries were sequenced with the Illumina HiSeq X Ten platform. The sequence of the primers used for specific amplification of guide barcodes is listed in Table S7 (Supporting Information).

### PacBio full‐length Transcriptome Sequencing Analysis

SMRTbell libraries were constructed and sequenced on the PacBio Sequel system. A total of five PacBio cells were performed for transcriptome sequencing in the K562 cell line. Initial data analysis was performed with Iso‐Seq 3.1.0 protocol (https://github.com/PacificBiosciences/IsoSeq). In the step1, raw subreads were converted to circular consensus (CCS) reads by “ccs” tool with the parameter “–noPolish –minLength = 300 –minPasses = 1 –minZScore = −999 –maxDropFraction = 0.8 –minPredictedAccuracy = 0.8 –minSnr = 4”, and removed primer sequence by “lima” tools. The FLNC reads, which contained 5′, 3′ primer and poly(A) tail, were generated by “isoseq3 refine” tool. In the step2, to avoid reads redundancy and improve reads accuracy, “cluster” tool was used on transcripts supported by >1 FLNC reads, and the high‐quality transcripts were generated after error correction by “polish” tools. In order to obtain exhaustive transcripts, the high‐quality transcripts were combined and transcripts supported by only one FLNC for the following analysis. In the step3, unique FLNC reads were mapped to Homo sapiens genome (Ensembl GRCh38.p12) by GMAP^[^ ^41^ ^]^ with the parameters “–trim‐end‐exons 12 –min‐trimmed‐coverage 0.8 –min‐identity 0.8 –min‐intronlength 70 ‐K 50 000”, 94% unique FLNC reads mapped to the human genome. In step4, the unique mapping bam files were then processed using the “collapse” module (collapse_isoforms_by_sam.py) of Cupcake software (https://github.com/Magdoll/cDNA_Cupcake) to generate non‐redundancy PacBio isoform (PB isoform) model with the parameters “‐c 0.8 ‐i 0.8 –dun‐merge‐5‐shorter”, which merges transcripts based on genomic coordinates.

Then, we used the SQANTI2^[^ ^42^ ^]^ and GffCompare^[^ ^43^ ^]^ software to categorize each PB isoform according to the reference transcript on Ensembl 92 version. Isoforms of annotated genes (protein‐coding and lncRNA) were detected based on shared splicing sites with reference, both novel and annotated isoforms were counted in Table S1 (Supporting Information). The isoforms of annotated genes were the transcript with “FSM”, “ISM”, and “NIC” structural categories of SQANTI2, and the transcripts both with the “NNC” categories of SQANTI2 and “c”, “k”, “m”, “n”, “j” class code of GffCompare. The class codes of two software underlying transcript classification were: 1) structural category of SQANTI2: “FSM”, full splice match; “ISM”, incomplete splice match; “NIC”, novel in catalog; “NNC”, novel not in the catalog. 2) class code of GffCompare: “c”, contained in reference; “k”, containment of reference; “m”, retained intron(s), all introns matched or retained; “n”, retained intron(s), not all introns matched/covered; “j”, multi‐exon with at least one junction.

Based on the PB isoforms generated by initial data analysis, we identified potential molncRNA sets with the following steps: 1) obtained the PB isoforms that overlapped with pre‐miRNA on genomic coordinates by BEDTools,^[^ ^44^ ^]^ and the transcription direction was consistent. 2) filtered out the isoforms that were annotated as “protein‐coding” genes with “FSM”, “ISM”, “NIC”, and “NNC” structural categories by SQANTI2. 3) evaluated the protein‐coding potential of PB isoforms using CPC2^[^ ^45^ ^]^ and CPAT^[^ ^46^ ^]^ software with default parameters. The non‐coding PB isoforms were retained with a recommended cut‐off <0.364 of CPAT and the “noncoding” flag of CPC2 software. 4) PB isoforms with length> 100 kb, length< 200 bp, or spanning annotated gene loci >5 were excluded. Further, the molncRNAs were classified into two types, molncRNAs contained pre‐miRNA sequences within exons/introns were named ex‐molncRNA / in‐molncRNA. The molncRNA was named using the embedded miRNA number, e.g., molnc‐22 for “*MIR22* originated lncRNA”. Where there were several miRNA genes embedded by the same molncRNA, the molncRNA was named after the 5′ end miRNA. In addition, we appended “cluster” after the name of molncRNA covering multiple miRNAs, when this name was the same as molncRNA covering with single miRNA. Isoforms of every molncRNA were numbered in Table S1, Supporting Information) (molnc_1, 2, 3…).

The positional relationship between molncRNAs and nearby annotated genes was categorized based on the structural category of SQANTI2, molncRNAs were classed into intragenic (molncRNA sharing loci with annotated genes on the same strands), antisense (molncRNA sharing loci with annotated genes on the opposite strands) and intergenic RNAs (related to Figure S1B, Supporting Information). Further, 786 molncRNAs located in the intragenic regions were classified into three subcategories based on the shared spliced sites between molncRNA and annotated gene: full splice match (FSM), partial splice match (PSM), complete splice mismatch (CSM).

Expression of full‐length RNA identified by Iso‐seq was counted by “get_abundance_post_collapse.py” in Cupcake software. The genomic visualization of molncRNA sequence was generated by IGV software.^[^ ^47^ ^]^ The conservation analysis of molncRNAs and annotated genomic elements were based on the phastCons scores across 100 species on UCSC database.^[^ ^48^ ^]^ The average phastCons score of the location of molncRNAs and genomic elements were calculated by bigWigAverageOverBed.^[^ ^49^ ^]^

### ChIP‐seq Datasets Analysis

The ChIP‐seq datasets of histone modification (H3K4me3, H3K27ac, H3K36me3) and Pol II in K562 cells were downloaded from the European Bioinformatics Institute (http://wwwdev.ebi.ac.uk/).^[^ ^50^ ^]^ Sources of these data are listed in Table S7 (Supporting Information). For overall ChIP‐seq datasets, reads were aligned to the Homo sapiens genome (Ensembl GRCh38.p12) using Bowtie2 with default parameters. Only reads with mapping quality scores>30 were retained for further analysis by SAMtools.^[^ ^51^ ^]^ Two biological replicates were merged to create the “Tag Directory” file by HOMER “makeTagDirectory”.^[^ ^52^ ^]^ The histone modification and Pol II ChIP fragment depth around the 5′ end of molncRNA isoform were made by HOMER “annotatePeaks.pl”.^[^ ^52^ ^]^

### Small RNA Datasets Analysis

Libraries were barcoded and subjected to 50‐bp pair‐end deep sequencing on the Illumina MiSeq platform by Novogene (Tianjin, China). Reads were aligned to the Homo sapiens genome (Ensembl GRCh38.p12) using Bowtie^[^ ^53^ ^]^ with default parameters. miRbase v21 annotation was used to count miRNA expression using featureCounts.^[^ ^54^ ^]^ Only reads with mapping quality scores>30 were retained for further analysis by SAMtools.^[^ ^51^ ^]^

### poly(A)+ RNA‐seq Datasets Analysis

Libraries (*n* = 2 biological replicates) were barcoded and subjected to 150‐bp pair‐end deep sequencing on the Illumina X Ten and NovaSeq platform by Novogene (Tianjin, China). Analysis of RNA‐seq datasets, reads were aligned to the Homo sapiens genome (Ensembl GRCh38.p12 in Figures 1 and 2; Figures S1 and S2 (Supporting Information), Ensembl GRCh38.p5 in Figure 5; Figure S5, Supporting Information) using Tophat2^[^ ^55^ ^]^ PE mode with the default parameter, and uniquely mapped reads were retained for further analysis filtered by SAMtools.^[^ ^51^ ^]^ HTSeq‐count^[^ ^56^ ^]^ or featureCounts^[^ ^54^ ^]^ were used for calculating annotated gene counts. Differential gene expression analysis was conducted using DESeq2^[^ ^57^ ^]^ with a *p*‐value <0.05, and all genes with non‐zero counts in the samples were considered. Raw counts were then normalized using DESeq2.

Analysis of molncRNA expression, short reads were aligned to molncRNA transcripts generated by Iso‐seq using Bowtie2^[^ ^58^ ^]^ with the parameters: “–no‐mixed –no‐discordant –no‐unal –end‐to‐end ‐k 200”. To avoid the influence of a high abundance of protein‐coding genes on molncRNA counts, transcripts that overlapped > 500 nt with annotated protein‐coding genes were excluded (Figures 1 and 2; Figures S1 and S2, Supporting Information). Only reads with mapping quality score >1 were retained for further analysis by SAMtools.^[^ ^51^ ^]^ Raw counts were calculated by in‐house script, the abundance of each molncRNA was sum of high‐quality unique mapped short fragments that were aligned on the molncRNA transcript. For multiple‐mapped fragments, the counts were added up to all mapped transcripts equally. Raw counts were then normalized using DESeq2. The expression correlation between molncRNA and the cognate miRNAs was evaluated by Pearson correlation analysis during human erythroid differentiation (days 11, 14, 18).

Expression of molncRNAs in 13 distinct blood cell populations^[^ ^21^ ^]^ and 16 human tissues (Human Body Map 2.0 Project) were based on RNA‐seq datasets (*n* = 2 biological replicates) from published papers, details of these data sources were listed in Table S7 (Supporting Information). The “combat” function in the SVA R package^[^ ^59^ ^]^ was applied to remove batch effects. Normalized counts of molncRNA were generated by DESeq2. The tissue and lineage‐specific molncRNAs were identified by the “ROKU” function in TCC R package^[^ ^60^ ^]^ with the parameter “upper.limit = 0.25”.

Dynamic expression of molncRNAs during erythroid (day 11, 14, 18), granulocytic (day 5, 10, 15),^[^ ^61^ ^]^ or monocytic (day 5, 10, 15)^[^ ^61^ ^]^ differentiation were classified by comparing normalized counts among three stages. The details of granulocytic and monocytic differentiation RNA‐seq datasets were listed in Table S7 (Supporting Information) “Continuously down” and “Continuously up” path represents that a molncRNA was down‐/up‐regulated (Fold‐change > 1.5 or Fold‐change < 0.67 and adjusted *p*‐value! = NA) between adjacent two stages. The “up‐down” path represents that a molncRNA was up‐regulated at day 14 compared with day 11 while down‐regulated at day 18 compared with day 14.

For RNA half‐life profiling, the ERCC sequence was downloaded from the Thermofisher website (https://www.thermofisher.com). DESeq2 was used to calculate ERCC spike‐in RNA size factors, which were then applied to normalize library size in each replicate. According to the quintile of mRNAs' half‐lives, RNAs were divided into five intervals as Q1, Q2, Q3, Q4, and Q5, ranking from long to short half‐life.

### MS Datasets Analysis

The MS spectrometry data was analyzed by Proteome Discoverer 1.4 (Thermo Fisher Scientific) supported by the Center of Biomedical Analysis (Tsinghua University, Beijing, China). The protein score, which was used to estimate the reliability of protein, was evaluated by PSMs (peptide spectrum matches – the number of identified peptides spectra matched for the proteins in second‐order MS), matching rate, and peptide FDR confidence level. For analysis of MS data generated from molnc‐301b RNA pull‐down assay in Figure 5, the molnc‐301b interacting proteins with score >5 were filtered and filtered out Keratin proteins. Locations of interacting proteins were classified according to the “subcellular location” section in Uniprot^[^ ^62^ ^]^ database (https://www.uniprot.org/). The GO functional network of nuclear protein contained in chromatin‐associated biological processes was generated by Cytoscape.^[^ ^63^ ^]^

### ChIRP‐seq Datasets Analysis

Libraries (*n* = 2 biological replicates) were barcoded and subjected to 150‐bp pair‐end deep sequencing on the Illumina X Ten platform by Novogene (Tianjin, China). For both IP and Input datasets, reads were aligned to reference genome Homo sapiens genome (Ensembl GRCh38.p5) using Bowtie2.^[^ ^58^ ^]^ Only reads with mapping quality scores>30 were retained for further analysis by SAMtools.^[^ ^51^ ^]^ And per‐base coverage was normalized in accordance with previous studies.^[^ ^64^ ^]^ For each base pair of the genome, the true coverage of this base in this sample was defined as the minimum coverage of the even lane and odd lane, and a SAM file was generated for peak calling. Peaks of each sample were then called using MACS2^[^ ^65^ ^]^ against its corresponding input with the following configurations: “–bw 250 ‐m 10 50 ‐g hs ‐p 0.00001 –nomodel –extsize 100”. The raw data profile was filtered for peaks with substantial correlation, and high coverage across the peaks was accepted. For the ChIRP‐seq sample, thresholds of average coverage >0.25, fold enrichment >5, and fold differences >1, the peaks overlapped with LacZ peaks were also removed. These peaks were annotated by HOMER^[^ ^52^ ^]^ and the enrichment of RNA binding sites on the genomic region was calculated by HOMER “annotatePeaks.pl”. The genomic visualization of molnc‐301b ChIRP‐seq datasets at the indicated gene locus was generated by IGV software.^[^ ^47^ ^]^

### Ribo‐seq Datasets Analysis

Libraries (*n* = 2 biological replicates) were barcoded and subjected to 150‐bp pair‐end deep sequencing on the Illumina X Ten platform by Novogene (Tianjin, China). Raw reads were trimmed by Cutadapt^[^ ^66^ ^]^ with parameters “‐e 0.1 ‐O 5 ‐n 2”. Then, reads with distinct inline barcodes were demultiplexed using in‐house scripts, and the 10‐mer random sequence was appended to the reads name in bam file for later usage. Low‐quality reads were filtered by Trimmomatic^[^ ^67^ ^]^ with the parameter “SLIDINGWINDOW: 4:15”. In Ribo‐seq library, reads length in 25–50 bp were retained for subsequent analysis. rRNA reads were removed by aligning reads with the rRNA reference sequence on NCBI database by Bowtie.^[^ ^53^ ^]^ Cleaned reads were mapped to Homo sapiens genome (Ensembl GRCh38.p5) by STAR.^[^ ^68^ ^]^ Uniquely mapped reads with “NH: i:1” flag were retained for further analysis by SAMtools^[^ ^51^ ^]^ and PCR duplicate reads were removed by in‐house script based on sharing identical random sequences. To quantify gene expression and translation abundance, sequenced reads mapping to CDS region were counted by featureCounts.^[^ ^54^ ^]^ DTEG tools^[^ ^69^ ^]^ were used (DESeq2‐based) to estimate TE, which was calculated by taking the ratio of Ribo‐seq over RNA‐seq counts. A gene was considered differentially translated when it met *p*‐value <0.05.

This technique enabled us to capture the fundamental features of translation, of which the distribution of ribosome‐protected mRNA fragments (RPFs) read lengths peaked at approximately 30 nt, and RPF density was highest at the start and stop codons. In contrast, mRNA‐seq tags were mapped uniformly across the length of the mRNA. Moreover, the offset between the 5′ terminus of an RPF and the ribosome P site was 13 nt (Figure S6D, Supporting Information). The vast majority of RPFs mapped to annotated CDSs (Figure S6F, Supporting Information). Additionally, in sharp contrast to the 5′ termini of the RNA‐seq tags, which mapped to all three codon nucleotides equally, the RPF termini mapped predominantly to the first nucleotide of the codon (Figure S6G, Supporting Information). The unique mapping reads in Ribo‐seq and RNA‐seq were used to analyze the 3‐nt periodicity, P‐site calibration, and meta‐gene read depth by Plastid.^[^ ^70^ ^]^ Length of RPFs and their distribution on CDS, 5′ UTR and 3′ UTR region of annotated protein‐coding genes were performed by Riboprofiling^[^ ^71^ ^]^ software. The repeatability between two biological replicates was evaluated by the Pearson correlation coefficient with RPF and mRNA counts on the CDS region.

### Gene Ontology Functional Enrichment Analysis

Gene ontology functional enrichment analysis was performed by “enrichGO” function in clusterProfiler R package,^[^ ^72^ ^]^ and it was mainly focused on “biological process”. Terms with *p*‐value<0.05 were considered to be enriched.

### scRNA‐seq Dataset Analysis

For the design of gRNA library, whole pre‐miRNA loci (1881, miRbase v21) were filtered according to the following rules, which accorded to the comparison of the relative positions of pre‐miRNA and annotated genes on genomic coordinates. 1) pre‐miRNA was located in the intergenic region without annotated ncRNA in −10 kb to 10 kb or protein‐coding genes in −8.5 kb to 8.5 kb region; 2) pre‐miRNA was completely overlapped with ncRNA or within 1 kb of ncRNA on the same strand and was not overlapped with annotated protein‐coding genes in −8.5 kb to 8.5 kb region. Finally, 361 pre‐miRNAs were filtered out for the following gRNA design.

Three replicates were applied for CROP‐seq. All 10x scRNA‐seq datasets were processed with the Cell Ranger (v3.0.2) pipeline (https://www.10xgenomics.com/) and were mapped to the Homo sapiens genome (Ensembl GRCh38.p12). This step yields a total of 258505 cells, and an average of 43105 reads per cell; an average of 2600 genes per cell, and an average 13357 of UMIs per cell.

Libraries of gRNA amplification were separately indexed and sequenced as spike‐ins alongside the scRNA‐seq libraries. UMI and cell‐barcode assignments were made for each read by processing these samples with the Cell Ranger (v3.0.2) “count” tools. Then, the output bam files and the whitelist of guides sequence were combined in the libraries to obtain the cell‐barcode and guide pairwise using “get_barcodes.py”^[^ ^33^ ^]^ with parameters: “–search_seq TTGTGGAAAGGACGAAACACCG –all_reads –barcode_length 20 –no_swalign –chimeric_threshold 0.2”. The similar chimeric sequences and reads unassigned to a gRNA were removed. Cell barcodes detected in the gRNA amplification libraries were then matched with cell barcodes detected in the regular 10× scRNA‐seq libraries. The gRNA with the largest UMI value was defined as the dominant gRNA.

Next, cells with unique gRNA (56509 cells) were retained and used R package Seurat^[^ ^73^ ^]^ for downstream analysis. 500 cells in each dataset are randomly selected as a NTC. All samples were aggregated and single‐cell UMI count was normalized with “SCTransform” function in Seurat. High‐quality cells (52434 cells) with >800 detected genes, >5000 total UMI counts, <25% of mitochondrial UMI counts, and <50% of ribosome UMI counts were obtained. For clustering, principal component analysis (PCA) was performed for dimension reduction. The top 12 principal components were selected using a permutation‐based test implemented in Seurat and passed to UMAP for clustering visualization. The top 50 genes in PC1 and PC2 were applied to gene functional enrichment analysis by Metascape.^[^ ^74^ ^]^ The phenotypic similarity between different gRNAs targeting the same gene was shown by hierarchical clustering of gene expression. Further, the proportion of gRNA was calculated whose node number spaced between any two gRNAs targeting the same molncRNA was <20% of total gRNAs. In the opinion, close clustering indicated similar phenotypes. To detect the reliable transcriptome perturbation after IC gene knockdown, those IC gRNA‐targeted cells were selected whose IC gene expression values were lower than the average value in NTC cells. Genes expression value was normalized by “SCTransform” function in Seurat. Differential gene expression analysis was performed between each gene knockdown group (cell with molncRNAs or IC gRNAs) and NTC group (cells without gRNAs) using the R package DESeq2 treating each cell as one replicate, and the batch effect was removed according to replicate batch. The top 100 most significantly altered genes were selected for each gene in IC+ or IC‐ group and merged together to form the signature DEGs list, and removed overlapped DEGs in both groups, which might merely reflect the phenotype triggered by gRNA infection.

The similarity of transcriptome changes was calculated based on the ratio of overlapping DEGs number and total DEGs number between molncRNAs‐targeted cells and IC+ or IC‐ genes‐targeted cells. Every *molncRNA* gRNA had two similarity values, calculated between molncRNAs‐targeted cells and IC+ genes‐targeted cells or molncRNAs‐targeted cells and IC‐genes‐targeted cells. The cutoff defined by the plus and minus one standard deviation around the means of similarity ratio (similarity _NTC, IC‐_ / similarity _NTC, IC+_), the threshold is 0.57 to 1.43. Based on the new cutoff, molncRNA gRNAs were considered as having the potential to promote erythroid differentiation if its similarity _molnc, IC+_ was 1.75‐fold greater than with similarity _molnc, IC‐_. The molncRNA gRNAs were considered as having the potential to inhibit erythroid differentiation if its similarity _molnc, IC‐_ was 1.43‐fold greater than with similarity _molnc, IC+_. Finally, the functional gRNAs in *molncRNA* ^(‐)^ and *molncRNA* ^(‐/‐)^ libraries were compared, and divided molncRNAs and miRNAs into four categories: i) same, lncRNA and miRNA have similar phenotypes; ii). different, lncRNA and miRNA have opposite phenotypes; iii) molncRNA^(‐)^ only, lncRNA has functions independent of its cognate miRNA; iv. molncRNA^(‐/‐)^ only, miRNA has functions independent of molncRNA. And the molncRNAs hosting high confidence miRNAs were retinted, which conserved in mammals,^[^ ^75^ ^]^ disturbed specific biological processes upon CRISPR knockdown assays, or maximum(expression value) > 10 in human tissues (miTED database,^[^ ^76^ ^]^ the type of human tissues are same as in Figure S1L, Supporting Information).

### Quantification and Statistical Analysis

Statistical parameters are reported either in individual figures or corresponding figure legends. Quantification data are in general presented as bar/line plots, with the error bar representing mean ± SD, or boxplot, showing the median (middle line), first and third quartiles (box boundaries), and furthest observation or 1.5 times of the interquartile (end of whisker). Whenever asterisks are used to indicate statistical significance, *stands for *p* < 0.05, **p < 0.01, ***p < 0.001 and ****p < 0.0001. The n.a. represents “not available”, and ns represents “not significant”. All statistical analyses were done in R and GraphPad.

## Conflict of Interest

The authors declare no conflict of interest.

## Author Contributions

W.L., Y.H., and Y.R. contributed equally to this work. J.Y. and F.W. conceived the study; W.L. and Y.R. performed the experiments; Y.H. performed the bioinformatics analysis; M.H., Y.C., Y.W., and L.X. provided materials and reagents; C.H., S.L., K.W., Y.G., Y.S., Y.G., J.X., X.W., and Y.M. provided technical assistance; W.L. and Y.H. wrote the manuscript; J.Y. and F.W. revised the manuscript.

## Supporting information

Supporting Information

Supporting Information

Supporting Information

Supporting Information

Supporting Information

Supporting Information

Supporting Information

Supporting Information

## Data Availability

PacBio Iso‐seq datasets have been submitted to the NCBI SRA database under accession number: SRR15276560. Single‐cell RNA‐seq datasets have been deposited at the NCBI GEO database under accession number: GSE181338. Other raw sequencing datasets (small RNA‐seq, poly(A)+ RNA‐seq, ChIRP‐seq, RNA‐seq, and Ribo‐seq) are accessible through GEO accession number: GSE180565.
