# Supplementary material for: Deciphering the Functional Long Non‐Coding RNAs Derived from MicroRNA Loci
Source: Adv Sci (Weinh). 2023 Oct 17;10(33):2203987. doi: 10.1002/advs.202203987 (PMC10667839; doi:10.1002/advs.202203987)
Supplement: Supplementary file 1 — Supporting Information [file ADVS-10-2203987-s006.pdf]

ADVANCED SCIENCE
Open Access

Supporting Information

for *Adv. Sci.*, DOI 10.1002/advs.202203987

Deciphering the Functional Long Non-Coding RNAs Derived from MicroRNA Loci

Weiqian Li, Yue Huo, Yue Ren, Chenxi Han, Shuo Li, Kangning Wang, Manman He, Yiying Chen, Yanran Wang, Lingjie Xu, Yuehong Guo, Yanmin Si, Yufeng Gao, Jiayue Xu, Xiaoshuang Wang, Yanni Ma, Jia Yu and Fang Wang**

Supporting Information

Deciphering the functional long non-coding RNAs derived from microRNA loci

Weiqian Li, Yue Huo, Yue Ren, Chenxi Han, Shuo Li, Kangning Wang, Manman He, Yiying Chen, Yanran Wang, Lingjie Xu, Yuehong Guo, Yanmin Si, Yufeng Gao, Jiayue Xu, Xiaoshuang Wang, Yanni Ma, Jia Yu, Fang Wang**

This file includes:

Figure S1 to 8

Captions for Table S1 to 7

Figure S1

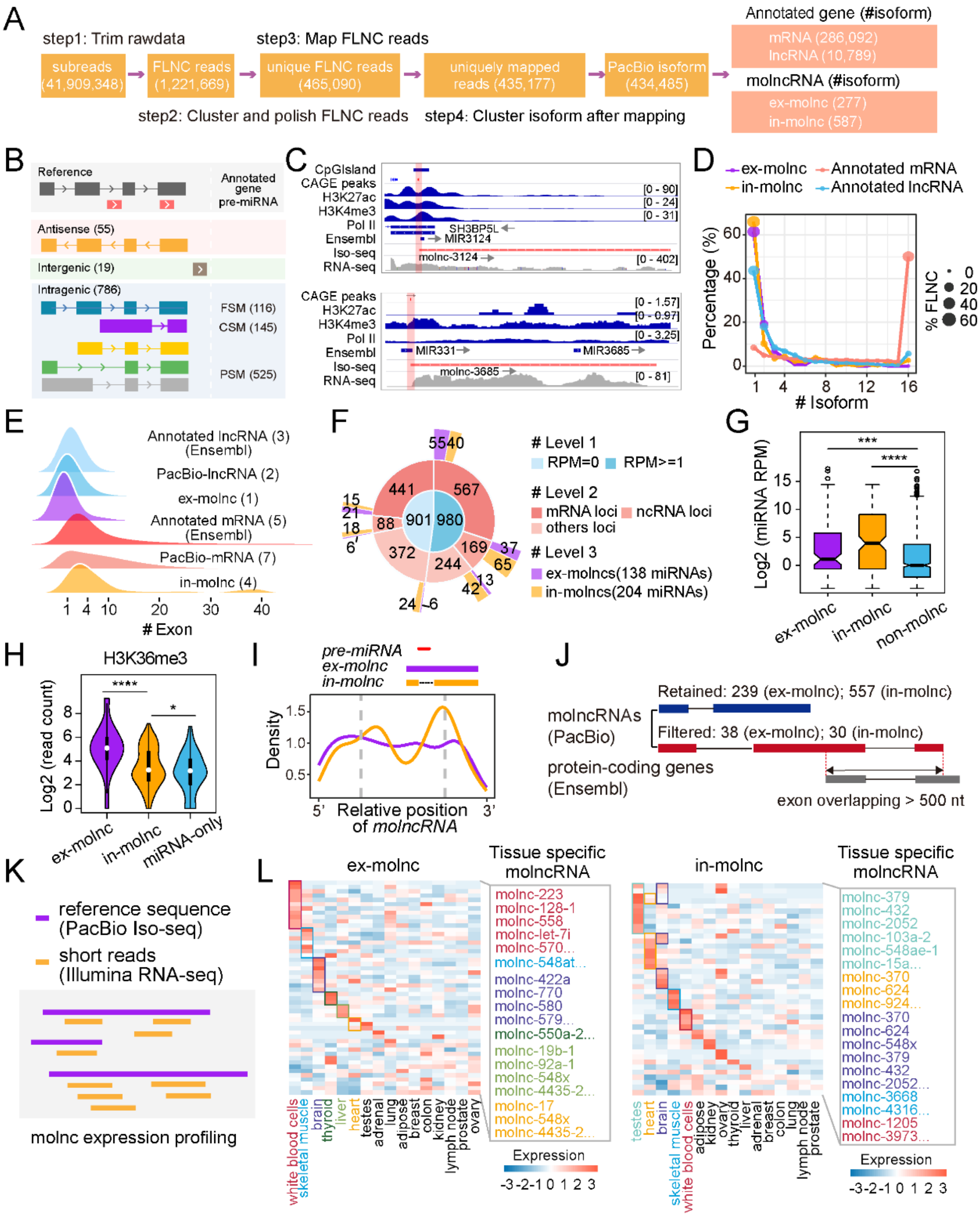

Figure S1. Characterization of molncRNAs based on PacBio Iso-seq and Illumina RNA-seq. Related to Figure 1. A) Workflow of PacBio Iso-seq analysis. **B**) Schematic illustration of antisense, intergenic and intragenic molncRNA. Arrows indicate transcription direction. MolncRNAs are classified into three categories based on the shared spliced sites between molncRNA and annotated gene: full splice match (FSM), complete splice mismatch (CSM), partial splice match (PSM). **C**) IGV plot of

identified antisense molnc-3124 and intergenic molnc-3685. The position of CpG island, CAGE peaks, the signals of H3K27ac, H3K4me3, Pol II ChIP-seq and reference sequence in Ensembl, gene model of full-length molncRNAs, as well as reads coverage generated from RNA-seq in hematopoietic cells, are displayed in order from top to bottom. **D**) Isoform number of molncRNA, Ensembl annotated mRNA and lncRNA. Circle size represents the percentage of RNAs with different isoform numbers. **E**) Exon number of molncRNA, mRNA and lncRNA. The median exon numbers are shown in brackets. **F**) Level 1: number of expressed and unexpressed miRNAs in K562 cells; Level 2: numbers of miRNAs embedded in annotated mRNA, ncRNA and other loci (the opposite strand of annotated genes or intergenic regions); Level 3: numbers of miRNAs embedded in molncRNAs. **G**) Boxplot showing expression levels of miRNAs derived from molncRNA and non-molncRNA loci (no molncRNAs were detected from the miRNA loci). p values were calculated by two-sided Mann-Whitney test. ***p <0.001, ****p <0.0001. **H**) The violin plot showing the H3K36me3 ChIP-seq read counts within -500 to 500 bp centered around pre-miRNAs in 'ex-molnc', 'in-molnc' and 'miRNA-only' group. p values were calculated by a two-sided Mann-Whitney test. *p <0.05, ****p <0.0001. **I**) Position of miRNA loci relative to molncRNA loci. For molncRNA, the thick lines represent exons and the thin lines represent introns. **J**) Schematic illustration of molncRNA filtering criterion: molncRNAs overlapping with annotated protein-coding genes by more than 500 nt were excluded from subsequent transcript count. The thick lines represent exons and the thin lines represent introns. **K**) Schematic illustration of Illumina poly(A)$^+$ RNA-seq mapping strategy. **L**) Heatmap of specific molncRNA expression across 16 distinct human tissues (n = 2 replicates).

Figure S2

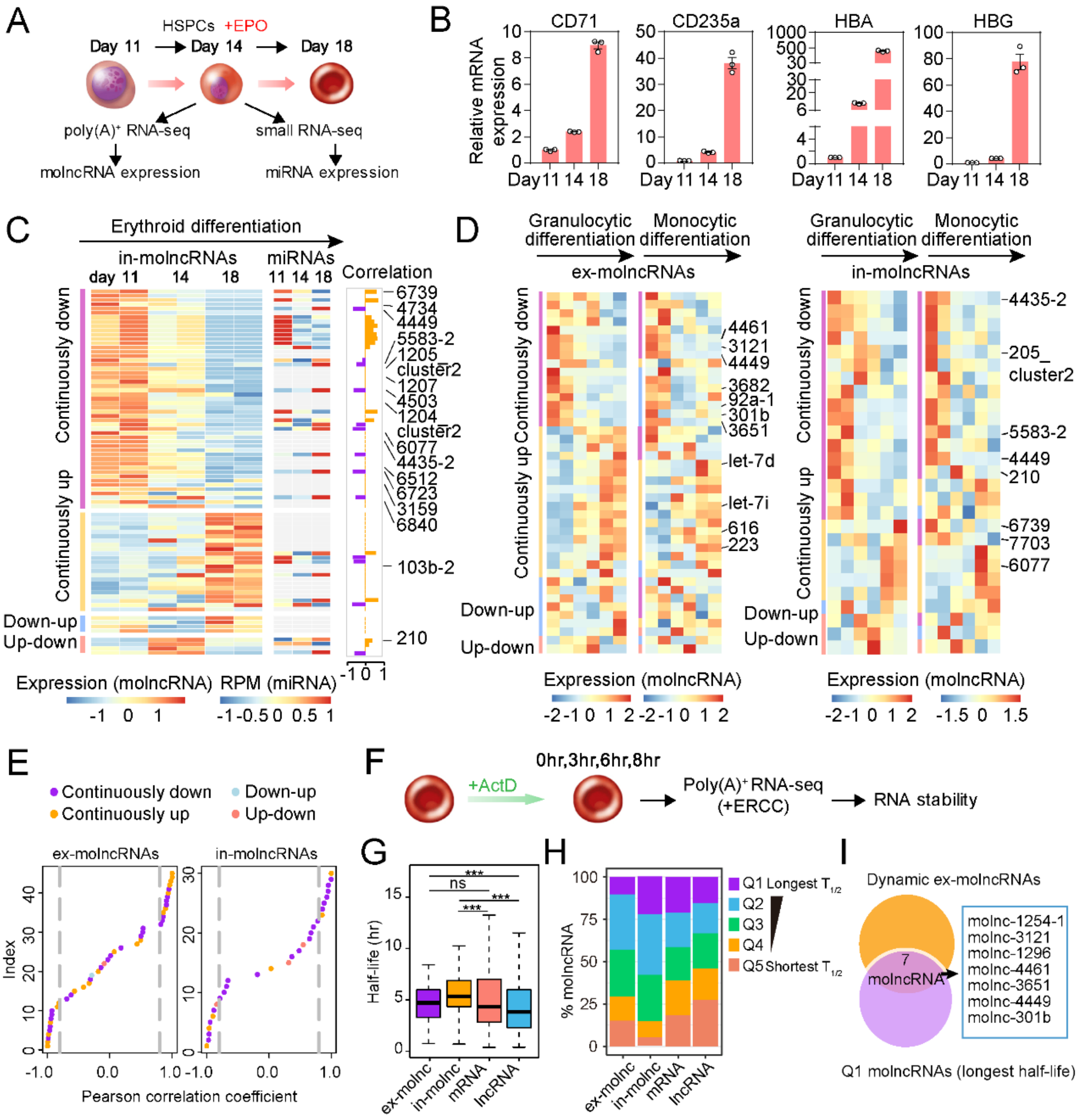

Figure S2. Dynamic molncRNA expression during human hematopoiesis. Related to Figure 2. A) Overview of RNA-seq sample collection. Erythroid differentiation of HSPCs was induced and cells were collected at days 11, 14 and 18 for poly(A)$^{+}$ and small RNA-seq. **B**) qPCR analysis of the relative expression of CD71, CD235a, HBA and HBG mRNAs during HSPC erythroid differentiation. Data represent the mean ± SD (n = 3 replicates). **C**) Heatmap of differentially expressed in-molncRNAs (left panel, n = 2 replicates) and their cognate miRNAs (middle panel) during erythroid differentiation. Pearson correlation analysis of molncRNAs and their cognate miRNAs is displayed in the right panel. **D**) Heatmap of differentially expressed ex-molncRNAs (left panel) and in-molncRNAs (right panel) during human

granulocytic and monocytic differentiation (n = 2 replicates). **E**) Pearson correlation analysis of molncRNAs and their cognate miRNAs. Gray dashed lines indicate ± 0.8. **F**) Overview of RNA-seq sample collection from K562 cells for RNA stability profiling (n = 2 replicates). **G**) Boxplot showing half-lives of molncRNA, mRNA and lncRNA. p values were calculated by two-sided Mann-Whitney test (n = 2 replicates). ***p <0.001, ns, not significant. **H**) Percentage of RNA (molncRNA, mRNA and lncRNA) half-lives in the five intervals (Q1, Q2, Q3, Q4 and Q5) based on mRNA half-lives quintile, ranked from the longest (Q1) to the shortest (Q5) (n = 2 replicates). **I**) Venn diagram showing the intersection of the dynamic (Figure 2B) and most stable ex-molncRNAs in the Q1 interval (Figure S2H).

Figure S3

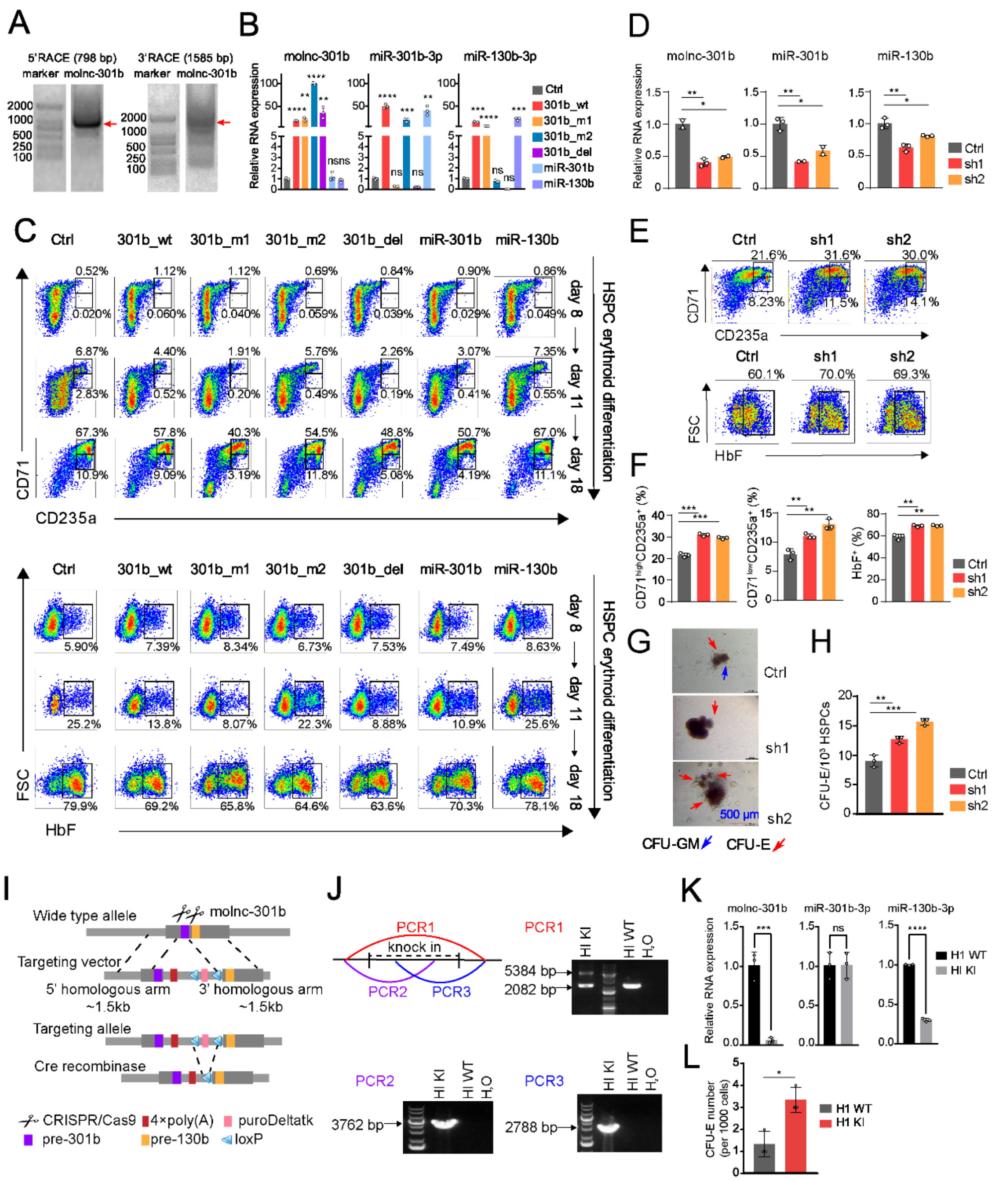

Figure S3. Pivotal role of molnc-301b during erythropoiesis. Related to Figure 3. **A**) Rapid-amplification of cDNA ends (RACE) of *molnc-301b* in K562 cells. **B**) qPCR analysis of molnc-301b, miR-301b and miR-130b expression in empty vector-(Ctrl) or molnc-301b construct-overexpressing HSPCs. Data represent the mean ± SD (n = 3 replicates). p values were calculated by unpaired *t*-test. **p <0.01, ***p <0.001, ****p <0.0001, ns, not significant. **C**) Representative flow cytometry plots of

$CD71^{high}/CD235a^{+}$, $CD71^{low}/CD235a^{+}$ and HbF^{+} cells in empty vector- (Ctrl) or molnc-301b construct-overexpressing HSPCs at the indicated days during erythroid differentiation. **D**) qPCR analysis of molnc-301b, miR-301b and miR-130b expression in empty vector- (Ctrl) or molnc-301b shRNAs-treated HSPCs. Data represent the mean ± SD (n = 3 replicates). p values were calculated by unpaired *t*-test. *p <0.05 and **p <0.01. **E)** Representative flow cytometry plots of $CD71^{high}/CD235a^{+}$, $CD71^{low}/CD235a^{+}$ and HbF^{+} cells in empty vector- (Ctrl) or molnc-301b knockdown HSPCs at day 11 during erythroid differentiation. **F)** Percentage of $CD71^{high}/CD235a^{+}$, $CD71^{low}/CD235a^{+}$ and HbF^{+} cells in empty vector- (Ctrl) or molnc-301b knockdown HSPCs after day 11 of erythroid differentiation detected by flow cytometry. Data represent the mean ± SD (n = 3 replicates). p values were calculated by unpaired *t*-test. **p <0.01 and ***p <0.001. **G)** Colony-forming unit assay of empty vector- (Ctrl) or molnc-301b knockdown HSPCs. Red arrows indicate colony-forming unit-erythroid (CFU-E) and blue arrows indicate colony-forming unit-granulocyte, macrophage (CFU-GM). Scale bar, 500 μm. **H)** Average frequency of colony formation per 10^3 HSPCs. Data represent the mean ± SD (n = 3 replicates). p values were calculated by unpaired *t*-test. **p <0.01 and ***p <0.001. **I**) Genomic editing strategy to generate miR-301b-4×poly(A) knockin H1 ESCs (HI KI). Primers (shown as red arrows) were designed within and outside of the editing region, and thus expected to generate bands of different sizes. **J**) PCR validation of the knockin of the *molnc-301b* locus. **K**) qPCR analysis of the relative expression of molnc-301b, miR-301b and miR-130b in H1 WT and HI KI hESCs. Data represent the mean ± SD (n = 3 replicates). p values were calculated by unpaired *t*-test. ***p <0.001, ****p <0.0001, ns, not significant. **L**) The quantification of erythroid progenitor (CFU-E) colonies generated from H1 KI and wild type hESCs, respectively. Data represent the mean ± SD (n = 3 replicates). p values were calculated by unpaired *t*-test. *p <0.05.

Figure S4

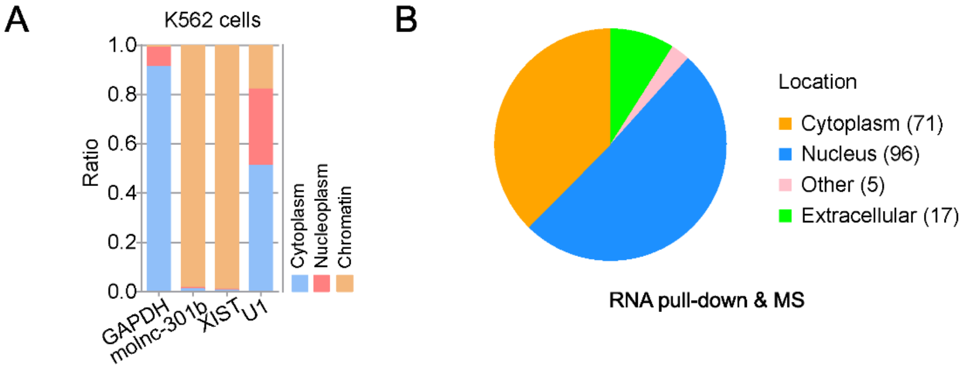

Figure S4. Molnc-301b evades the miRNA processing machinery. Related to Figure 4. A) Fractions located in the chromatin, nucleoplasm and cytoplasm of K562 cells. *GAPDH*, *U1*, XIST RNA were used as positive control for cytoplasm, nucleoplasm, chromatin gene expression, respectively (n = 3 replicates). **B**) RNA pull-down coupled with MS analysis showing subcellular localization of proteins interacting with molnc-301b. The number of proteins in each compartment is shown in brackets.

Figure S5

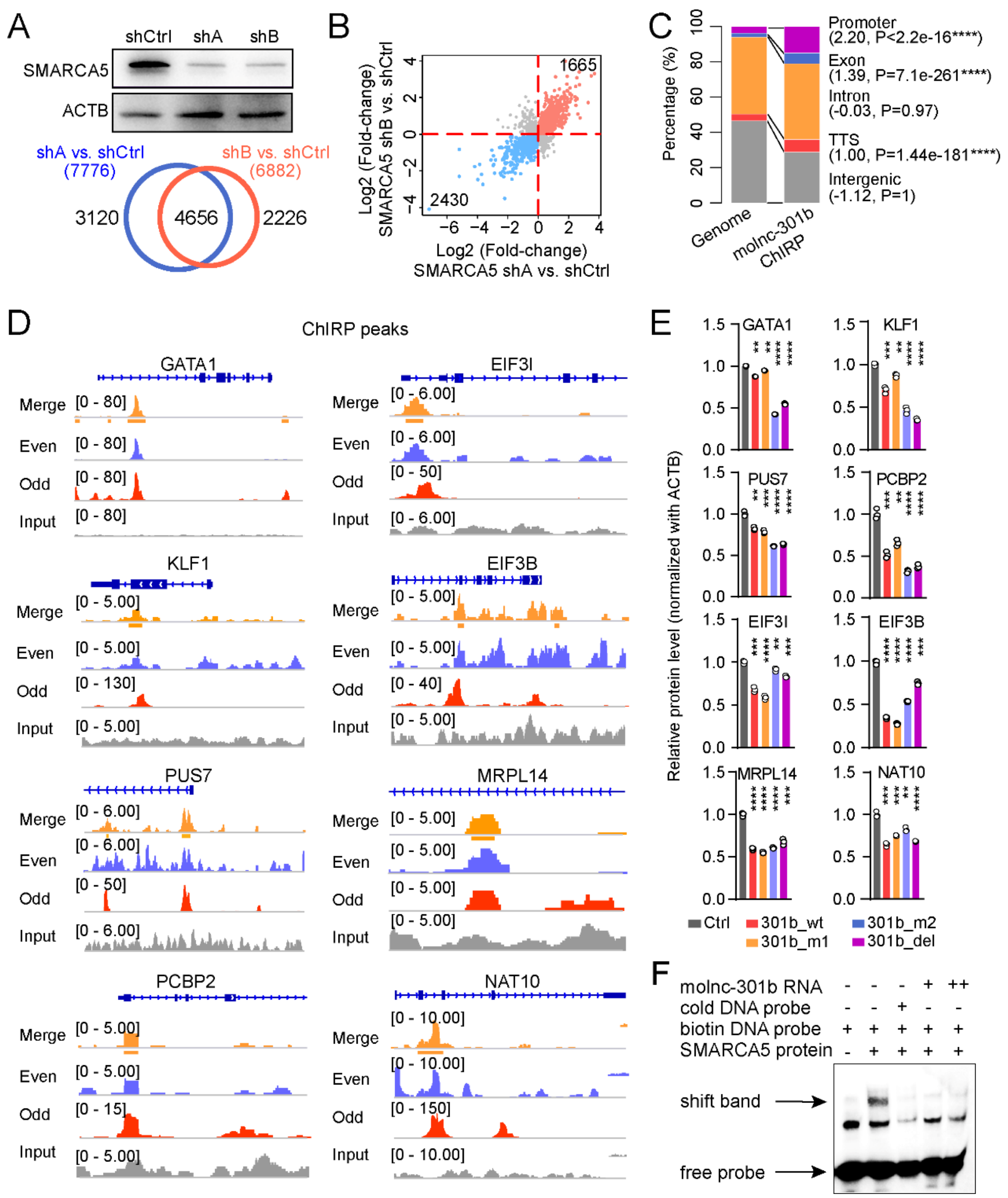

Figure S5. Molnc-301b binds to erythroid- and translation-associated gene loci. Related to Figure 5. A) Immunoblot analysis of SMARCA5 protein in K562 cells treated with SMARCA5-specific shRNAs (shA and shB) and scramble oligonucleotides (shCtrl) (upper panel). Venn diagram of DEGs between two SMARCA5 shRNAs (lower panel). **B**) Scatterplots showing DEGs of shA-, shB-treated cells compared with the shCtrl. Significantly up- or downregulated genes

were determined by $p < 0.05$ (n = 2 replicates). **C**) Enrichment of molnc-301b ChIRP-seq peaks in different genic and intergenic regions compared with the genome. The numbers in brackets indicate the log2 (enrichment ratio) relative to the genome (n = 2 replicates). p values were evaluated by single-tailed Fisher's exact test. ****$p < 0.0001$. **D**) Graphical representation of molnc-301b ChIRP-seq peaks among candidate gene loci. Merge lane represents the results of merged "even" and "odd" probe groups. The orange line represents the molnc-301b binding sites (n = 2 replicates). **E**) Quantitative analysis of protein levels from immune blots (Figure 5J). Data represent the mean ± SD (n = 3 replicates). p values were calculated by unpaired *t*-test. **<0.01, ***$p < 0.001$, ****$p < 0.0001$. **F**) *In vitro* association of SMARCA5 with DNA as identified by EMSA assay in which 5' biotin-labeled DNA probes were incubated with purified SMARCA5. The unlabeled DNA probe (cold DNA probe) was used in the competitive assays.

Figure S6

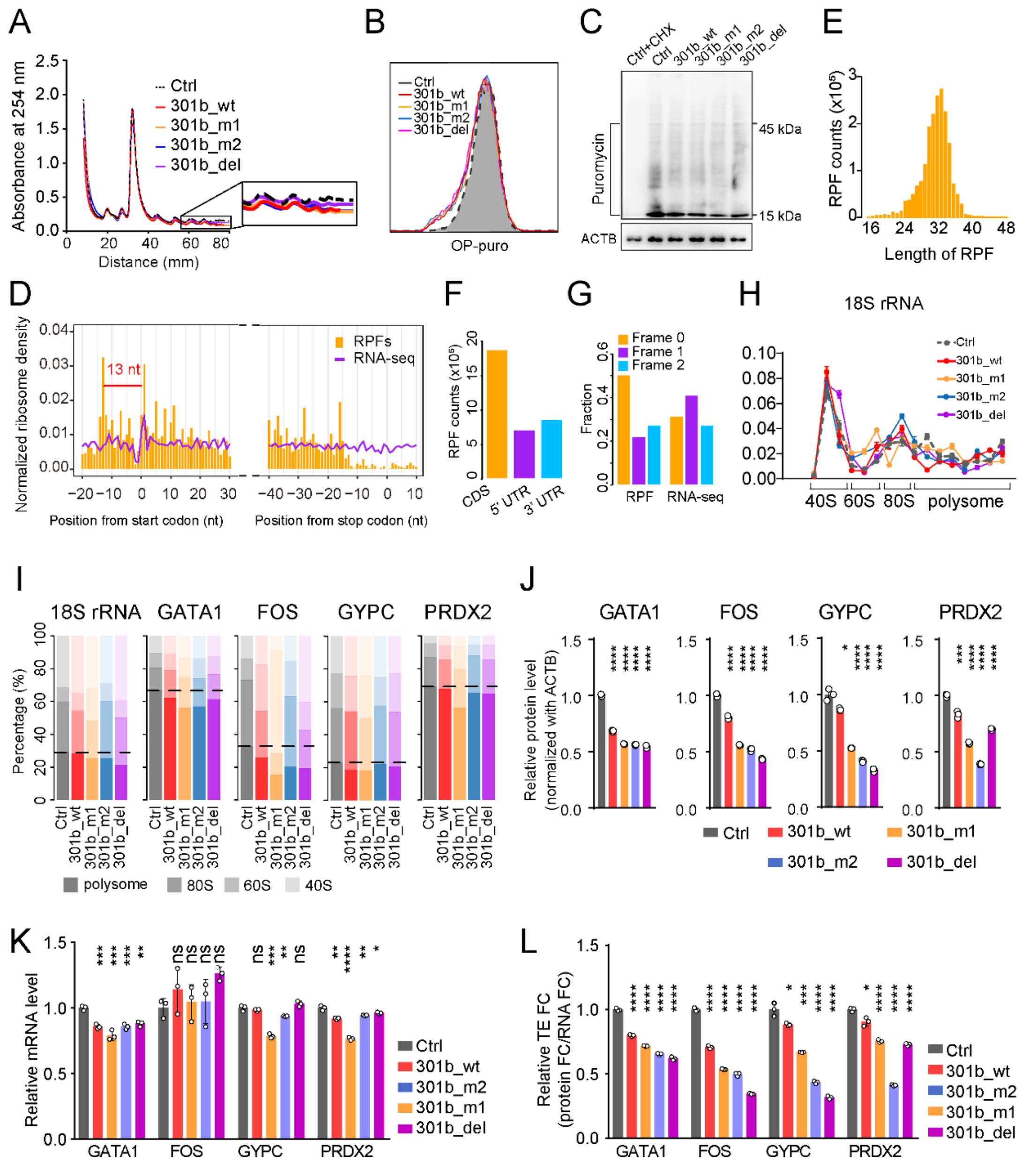

Figure S6. Quality control and data analysis of Ribo-seq. Related to Figure 6. A) Polysome profiles of K562 cells treated with empty vector- (Ctrl) or molnc-301b constructs and fractionated by sucrose gradient sedimentation. The relative absorbance of each component was then measured at 254 nm. **B)** OP-puro incorporation in K562 cells treated with Ctrl, 301b_wt, 301b_m1, 301b_m2 and 301b_del. **C)** SUnSET assays in K562 cells treated with empty vector- (Ctrl) or molnc-301b constructs. **D**) Density of RPFs and RNA tags near the ends of ORFs. The offset between the 5' terminus of an RPF and the first nucleotide in the human

ribosome P site is typically 13 nucleotides. **E**) Fragment length distribution of RPFs. **F**) RPFs were mainly distributed in the CDS region. **G**) Quality control of three codon nucleotides: most of the RPF started at the first nucleotide of a codon. **H**) Polysome RT-qPCR analysis of 18S rRNA in K562 cells treated with Ctrl, 301b_wt, 301b_m1, 301b_m2 and 301b_del (n = 3 replicates). **I**) Polysome RT-qPCR analysis of *GATA1*, *FOS*, *GYPC* and *PRDX2* transcripts in K562 cells treated with Ctrl, 301b_wt, 301b_m1, 301b_m2 and 301b_del. Dashed lines indicate the percentage RNA in the polysome fraction of K562 cells treated with Ctrl. **J**) Quantitative analysis of protein levels from immune blots (Fig 6G). **K**) RT-qPCR validation of four candidates in HSPCs. **L**) Relative TE fold-change (protein fold-change in Fig S6H/RNA fold-change in Fig S6I) of four candidates.

For Figure S6J-L, data represent the mean ± SD (n = 3 replicates). p values were calculated by unpaired *t*-test. *p < 0.05, **p <0.01, ***p <0.001, ****p <0.0001, ns, not significant.

Figure S7

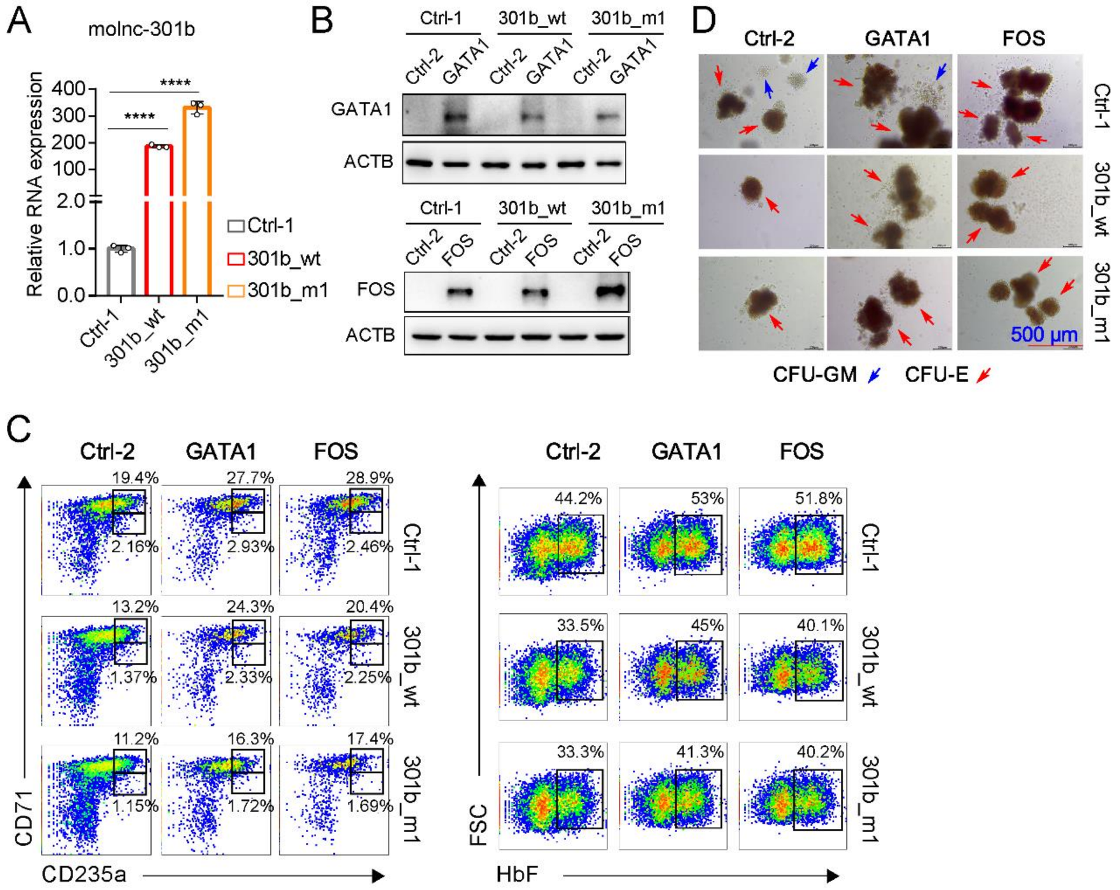

Figure S7. GATA1 and FOS are regulatory targets of molnc-301b during erythroid differentiation. Related to Figure 6. A) qPCR analysis of molnc-301b, in empty vector- (Ctrl-1) or molnc-301b-overexpressing HSPCs. Data represent the mean ± SD (n = 3 replicates). p values were calculated by unpaired *t*-test. ***p <0.0001. **B)** Immuno-blot of GATA1 and FOS in Ctrl-2- or GATA1/FOS-overexpressing HSPCs. **C)** Representative flow cytometry plots of CD71^{high}/CD235a^{+}, CD71^{low}/CD235a^{+} and HbF^{+} cells among HSPCs within Ctrl-1- or molnc-301b-treated populations, in which HSPCs were treated with Ctrl-1, wide type or mutant molnc-301b followed by Ctrl-2 or GATA1/FOS transduction. **D)** Colony-forming unit assay of Ctrl-1- or 301b_wt/m1-treated HSPCs, in which HSPCs were treated with Ctrl-1 or 301b_wt/m1 followed by Ctrl-2 or GATA1/FOS transduction. Red arrows indicate colony-forming unit-erythroid (CFU-E) and blue

arrows indicate colony-forming unit-granulocyte, macrophage (CFU-GM). Scale bar, 500 μm.

Figure S8

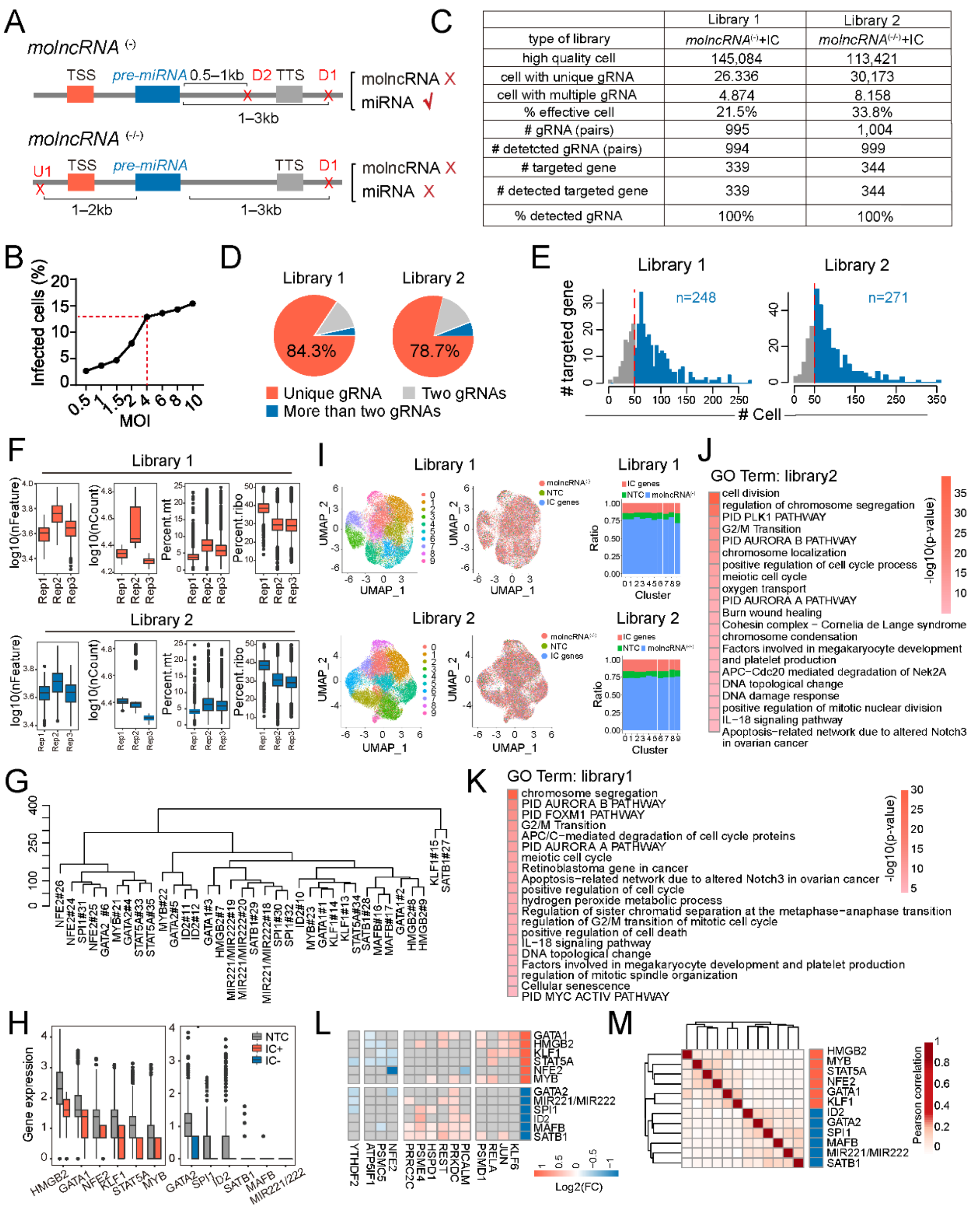

Figure S8. CROP-seq quality control and data analysis. Related to Figure 7. A) Schematic illustration of gRNA library construction strategy. **B)** Flow cytometric

analysis of cell infection by gRNA library virus. MOI, multiplicity of infection. **C**) Cell number and gRNA information of pooled CRISPR-Cas9-based screening with single-cell RNA sequencing in each library. **D**) Pie chart showing the proportion of cells expressing unique gRNA, two gRNAs, and more than two gRNAs in each library. The percentage of cells assigned to unique gRNAs is displayed. **E**) Histogram showing the molncRNAs- and IC genes-targeted cell number. **F**) Quality control of single-cell RNA-seq dataset after normalization and scale steps. **G**) The transcriptome clustering results of different gRNA groups. **H)** Boxplot showing the expression of internal control genes in IC-targeted cells or non-targeting control (NTC) cells. **I)** UMAP plots of all high-quality cells in library1 and library2. Left panel, the identified cell clusters are indicated by color. Middle panel, cells colored based on differential gRNA assignments. Right panel, UMAP plots of all high-quality cells. **J)** Gene functional annotation analysis of top 50 genes in PC1 and PC2 of library1. **K)** Gene functional annotation analysis of top 50 genes in PC1 and PC2 of library2. **L**) Heatmap displaying differential expression of erythroid-associated genes in cells with IC gRNAs. **M)** Heatmap showing Pearson's correlations between filtered DEGs genes among cells with IC gRNAs.

Table S1. Essential characteristic of molncRNAs, mRNAs and classic lncRNAs identified by PacBio Iso-seq and miRNA expression in K562 cells. Related to Figure 1, 2 and Figure S1, S2.

Table S2. Tissue and lineage specific molncRNA analyzed from published RNA-seq datasets. Related to Figure 1H and Figure S1L.

Table S3. Dynamic expression of molncRNA during human hematopoiesis and half-lives of molncRNA during erythroid differentiation. Related to Figure 2 and Figure S2.

Table S4. Differential expression genes in RNA-seq upon molnc-301b overexpression or RNA-seq upon SMARCA5 knockdown, molnc-301b MS dataset and co-regulated genes by molnc-301b and SMARCA5. Related to Figure 4, 5 and Figure S4, S5.

Table S5. Differential translation efficiency in Ribo-seq upon molnc-301b overexpression. Related to Figure 6 and Figure S6.

Table S6. gRNA information and analysis results in CROP-seq. Related to Figure 7 and Figure S8.

Table S7. Sequencing datasets and primers. Related to Figure 1-7 and Figure S1-8.
